# Fmp30p is a mitochondrial phosphatidylinositol hydrolase that modulates CoQ biosynthesis

**DOI:** 10.1038/s41467-026-73766-x

**Published:** 2026-05-30

**Authors:** Zakery N. Baker, Rachel M. Guerra, Sean W. Rogers, David J. Pagliarini

**Affiliations:** 1 Department of Cell Biology and Physiology, Washington University School of Medicine, St. Louis, MO USA; 2 Department of Biochemistry and Molecular Biophysics, Washington University School of Medicine, St. Louis, MO USA; 3 Department of Genetics, Washington University School of Medicine, St. Louis, MO USA; 4 Howard Hughes Medical Institute, Washington University School of Medicine, St. Louis, MO USA

**Keywords:** Membrane lipids, Mitochondria, Phospholipids

## Abstract

Organellar membranes feature bespoke lipid compositions; however, the enzymes that craft these compositions and the functional implications these lipids exert on membrane protein organization and activity are insufficiently understood. Here, we discover that the inner mitochondrial membrane protein Fmp30p, a member of the metallo-β-lactamase superfamily, displays phospholipase type D activity toward phosphatidylinositol (PI)—a notable mitochondrial membrane component with unclear functional roles. *FMP30* deletion caused substantial and specific elevation of PI species in purified mitochondria. Augmenting mitochondrial PI levels in this way, or by targeting established PI-modifying enzymes to the organelle, increased coenzyme Q (CoQ) biosynthesis concomitant with elevated expression of CoQ-related enzymes and enhanced CoQ metabolon formation. Collectively, our work establishes Fmp30p as a mitochondrial PI phospholipase related to CoQ biology and reveals the broader importance of inner membrane PI in regulating mitochondrial function.

## Abstract (web summary)

The mechanisms contributing to organellar membrane lipid composition remain unclear. Here, Baker and Guerra et al. characterize the mitochondrial protein Fmp30p as a phosphatidylinositol (PI) phospholipase-D enzyme that modulates PI abundance and links mitochondrial PI to coenzyme Q biosynthesis.

## Introduction

Coenzyme Q (CoQ) is an essential redox-active lipid that transfers electrons in the mitochondrial electron transport chain, acts as a key cofactor in biosynthetic processes, and prevents oxidative damage throughout the cell^1^. Nearly all organisms synthesize CoQ endogenously, with eukaryotic production occurring at the inner mitochondrial membrane^2^. Defects in CoQ biosynthesis cause diverse human pathologies, including ataxias and nephropathies^3^, and secondary CoQ deficiency is a common feature of numerous mitochondrial disorders^4^. Unfortunately, limited therapeutic options exist to ameliorate these deficiencies, as exogenous CoQ supplementation is hampered by its extreme hydrophobicity^5^. Elucidating mechanisms that regulate CoQ biosynthesis may motivate alternative therapeutic interventions that bolster endogenous CoQ production.

The critical nature of CoQ in cellular functions suggests that its synthesis and turnover may be subject to multiple levels of regulation, as is true for other bioactive lipids^6,7^. Early work linked the regulation of CoQ biosynthesis to lipid metabolism and oxidative stress responses via PPARα^8^ and NF-κB^9^, though the mechanisms remain unexplored and appear to be context- and species-specific. More recent studies have hinted at coordinated regulation of CoQ biosynthesis with other important mitochondrial processes, such as complex I assembly^10,11^, mitochondrial biogenesis^12^, and mitochondrial protein processing and import^13,14^. Moreover, a regulatory relationship between mitochondrial phospholipid metabolism and CoQ biosynthesis has been postulated^15^. However, we still lack a detailed understanding of how cells sense and integrate environmental or intracellular stimuli to modulate their CoQ content. As such, the CoQ regulatory landscape remains a ripe area for discovery with potential therapeutic implications.

In this study, we reveal a surprising link between CoQ abundance and the poorly characterized *S. cerevisiae* mitochondrial protein, Fmp30p. We discover that Fmp30p (Found in mitochondrial proteome 30; Ypl103c), a mitochondrial intermembrane space-localized protein belonging to the metallo-β-lactamase superfamily, is a lipid hydrolase with specificity toward phosphatidylinositol (PI). Furthermore, we reveal that modulation of mitochondrial PI content enhances CoQ metabolon formation and biosynthetic efficiency, and show that this relationship between PI and CoQ is conserved in mammals. Collectively, our data establish Fmp30p as a regulator of mitochondrial membrane phospholipid composition and provide an inaugural link between PI and a core mitochondrial process.

## Results

### *FMP30* deletion increases the rate of CoQ biosynthesis

**Fig. 1:**
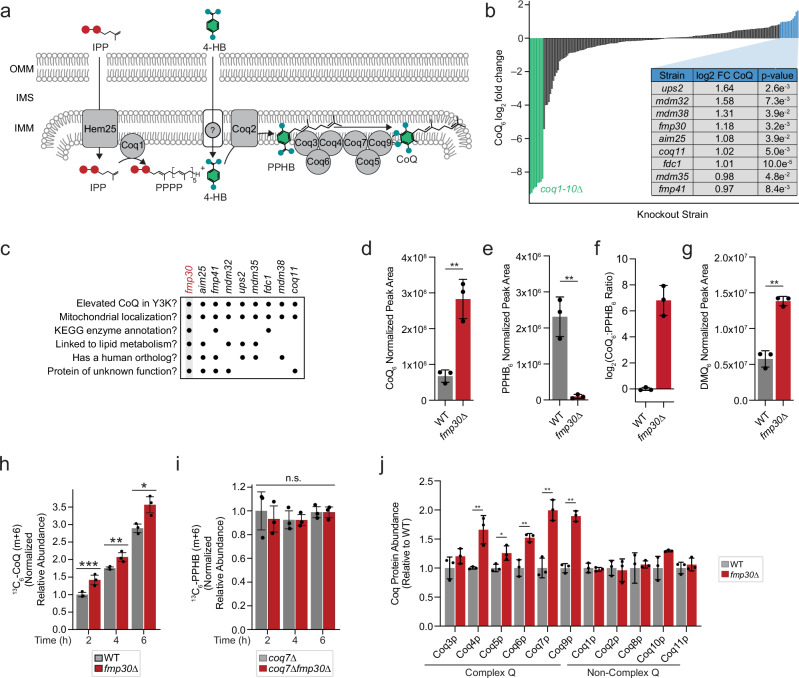
*FMP30* deletion increases CoQ biosynthetic machinery and de novo biosynthesis rates. **a** Schematic of CoQ biosynthesis in *S. cerevisiae*. Red circles represent phosphate groups, blue circles represent oxygen. IPP, isopentyl pyrophosphate; 4-HB, 4-hydroxybenzoate; PPPP, polyprenyl pyrophosphate; PPHB, polyprenyl-hydroxybenzoate; CoQ, coenzyme Q. **b** Rank ordering of gene deletion strains in the Y3K dataset^16^ based on their CoQ_6_ abundance. Strains with deletion in genes involved in CoQ biosynthesis are highlighted in green. Strains with increased CoQ abundance are highlighted in light blue. The inset chart lists the deletion strains from Y3K ranked by highest increase in CoQ abundance compared to wild type (WT). Log_2_ fold change derived from mean abundances of *n* = 3 biologically independent samples, *p*-value from two-sided Student’s *t* test. **c** Summary table of genes from Y3K with elevated CoQ abundance, analyzed for mitochondrial localization, putative enzyme activity, presence of a human ortholog, and known function. *FMP30* (highlighted) is the only gene to meet all criteria. **d** CoQ_6_ abundance in WT and *fmp30*Δ cells (**P = 2.92 × 10^−3^). **e** PPHB_6_ abundance in WT and *fmp30*Δ cells (**P = 2.29 × 10^−3^). **f** Log_2_ transformed ratio of CoQ_6_ to PPHB_6_ abundance in the *fmp30*Δ strain normalized to WT. **g** DMQ_6_ abundance in WT and *fmp30*Δ cells (**P = 4.39 × 10^−4^). **h** Abundance of de novo synthesized ^13^C_6_-CoQ_6_ in WT and *fmp30*Δ cells grown in –pABA SD 3% (wt/vol) glycerol and 50 μM [*phenyl*-^13^C_6_]−4-HB, relative to WT at 2 h (***P = 1.61 × 10^−2^, **P = 2.52 × 10^−2^, *P = 2.15 × 10^−2^). **i** Abundance of de novo synthesized ^13^C_6_-PPHB_6_ in *coq7*Δ and *coq7*Δ*fmp30*Δ cells grown in –pABA SD 3% (wt/vol) glycerol and 50 μM [*phenyl*-^13^C_6_]−4-HB (relative to *coq7*Δ at 2 h). **j** Relative protein abundance of complex Q proteins from *fmp30*Δ compared to WT. For **d**–**j** data, mean ± s.d. are displayed; *n* = 3 biologically independent samples; two-sided Student’s *t* test compared to WT samples. For analyses with more than four comparisons, *P < 0.05, **P < 0.01. Source data are provided as a Source Data file.

Coenzyme Q (CoQ) biosynthesis involves import of cytosolic precursors into the mitochondrial matrix, headgroup attachment to the polyisoprenoid tail to form polyprenyl-hydroxybenzoate (PPHB), and subsequent chemical headgroup modifications to form mature CoQ (Fig. 1a). To find potential regulators of this process, we searched for proteins that: 1) are mitochondrially localized, 2) have an enzymatic KEGG annotation, 3) lack a clear function, 4) possess a human ortholog, 5) have genetic or physical association to lipid metabolism, and 6) exhibit elevated CoQ abundance in our Y3K dataset—a collection of multiomic measurements from yeast strains lacking individual mitochondrial genes^16^ (Fig. 1b). We found one putative enzyme that met all these criteria (Fig. 1c), the understudied mitochondrial protein Fmp30p.

We set out to determine the mechanism underscoring the increased CoQ abundance in *FMP30* knockout cells. We first generated a fresh deletion of *FMP30* within the BY4742 background and measured levels of CoQ and its early and late biosynthetic precursors, PPHB and DMQ, respectively. Recapitulating our previous observations in the Y3K dataset, we found a significant increase in CoQ abundance in the *fmp30*Δ strain (Fig. 1d) matched by a decrease in PPHB levels (Fig. 1e). Together, the ratio of CoQ to PPHB was increased >20-fold over wild type (Fig. 1f), suggesting enhanced CoQ biosynthetic efficiency. We similarly observed an increase in DMQ (Fig. 1g), consistent with enhanced flux through the pathway in the absence of *FMP30*. To probe the rate of CoQ biosynthesis, we next measured de novo CoQ production using pulse labeling with ^13^C-labeled 4-hydroxybenzoic acid^17^ and found that deletion of *FMP30* increased de novo CoQ production at every timepoint examined (Fig. 1h). To correspondingly measure the rate of PPHB biosynthesis, we deleted *COQ7*, which thwarts CoQ biosynthesis beyond PPHB by disrupting complex Q—a metabolon comprised of CoQ biosynthetic enzymes^1^. *FMP30* deletion in this background did not change the rates of ^13^C-labeled PPHB generation (Fig. 1i), indicating that the increase in CoQ biosynthesis in *fmp30*Δ occurs at the headgroup modification stage (Fig. 1a).

Complex Q (aka the CoQ synthome) resides on the matrix face of the mitochondrial inner membrane^18,19^. We postulated that elevated complex Q protein abundance could significantly boost biosynthetic efficiency. The *fmp30*Δ strain exhibited a slight but statistically significant increase in nearly all complex Q members without affecting non-complex Q Coq proteins (Fig. 1j) or causing global changes to mitochondrial protein content (Supplementary Fig 1a). Among all strains in the Y3K dataset, *fmp30*Δ also possessed the second highest increase in complex Q proteins, further supporting Fmp30p’s connection to this pathway (Supplementary Fig 1b). We additionally performed mitochondrial crosslinking with affinity enrichment mass spectrometry (AEMS) on strains expressing tagged Coq9p to probe if new proteins were recruited to complex Q in the absence of *FMP30*. However, these results demonstrated that the composition of complex Q remains unchanged in *fmp30*Δ, despite the increased complex abundance (Supplementary Fig 1c, d). Furthermore, we saw no global alterations in mitochondrial morphology in *fmp30*Δ compared to wild type, including mitochondrial area or branching (Supplementary Fig 1e-h), as observed by fluorescence microscopy and TEM ultrastructure imaging (Supplementary Fig 1i). Collectively, these data suggest Fmp30p is a negative regulator of CoQ biosynthesis whose deletion augments complex Q abundance and leads to enhanced biosynthetic flux.

### Fmp30p is a PI hydrolase functionally distinct from human NAPE-PLD

**Fig. 2:**
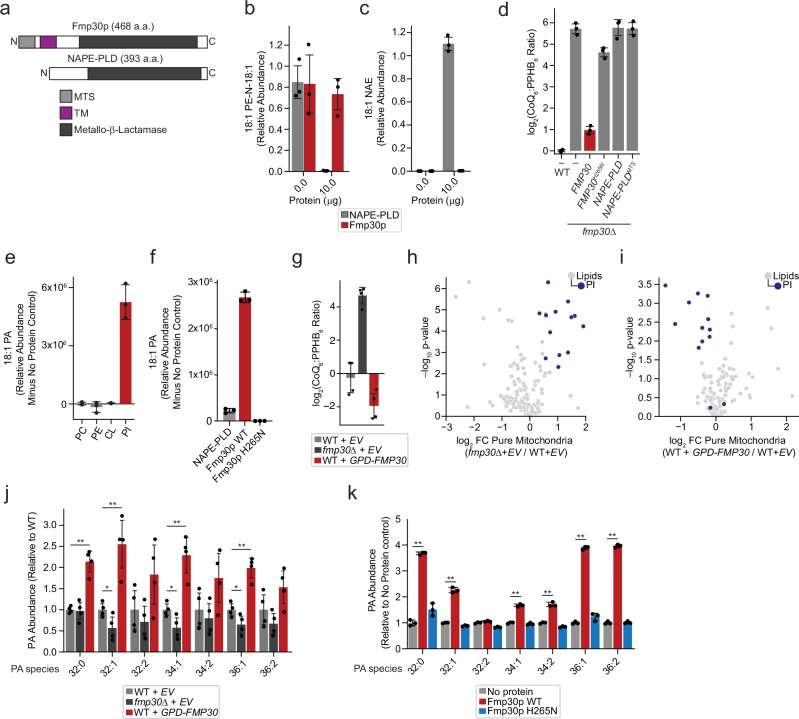
Fmp30p possesses phospholipase D activity toward phosphatidylinositol. **a** Diagram of Fmp30p and NAPE-PLD protein sequences highlighting protein domains. MTS, mitochondrial targeting sequence; TM, transmembrane domain. **b** Relative abundance of 18:1 PE-N-18:1 (18:1 NAPE) following incubation with the indicated concentration of purified recombinant Fmp30p or NAPE-PLD for 30 min. NAPE, N-acylphosphatidylethanolamine. **c** Relative abundance of 18:1 NAE generated after incubation of 18:1 PE-N-18:1 with the indicated concentration of purified recombinant Fmp30p or NAPE-PLD for 30 min. NAE, N-acylethanolamide. **d** Log_2_ transformed ratio of CoQ_6_ to PPHB_6_ abundance in WT or *fmp30*Δ strains expressing Fmp30p wild-type (*FMP30*), Fmp30p catalytically dead mutant (*FMP30 H265N*), NAPE-PLD wild-type (*NAPE-PLD*), or mitochondrially targeted NAPE-PLD (*NAPE-PLD*^*MTS*^), normalized to WT. **e** Relative abundance of 18:1 phosphatidic acid (PA) generated after incubation of the indicated phospholipid species with recombinant Fmp30p for 1 h. PC, phosphatidylcholine; PE, phosphatidylethanolamine; CL, cardiolipin, PI, phosphatidylinositol. **f** Relative abundance of 18:1 PA after incubation of 18:1 PI with recombinant NAPE-PLD, Fmp30p WT or Fmp30p H265N for 1 h. **g** Log_2_ transformed ratio of CoQ_6_ to PPHB_6_ abundance in pure mitochondria isolated from WT + empty vector (*EV)*, *fmp30*Δ + *EV*, and WT + *GPD-FMP30* yeast. Data normalized to WT + *EV*. Relative lipid abundance in purified mitochondria from *fmp30*Δ + *EV* (**h**) or WT with *FMP30* overexpression (*GPD-FMP30*) (**i**) compared to WT + *EV*, versus statistical significance. Phosphatidylinositol (PI) species are colored in dark blue. **j** Relative abundance of indicated phosphatidic acid (PA) species in pure mitochondria from WT carrying an *EV*, *fmp30*Δ carrying an *EV*, or WT overexpressing *FMP30* (*GPD-FMP30*). **k** Relative abundance of indicated phosphatidic acid (PA) species following incubation of pure mitochondria from *fmp30*Δ with no protein, recombinant Fmp30p WT, or Fmp30p H265N for 1 h. For **b**–**f** and **k**, data are displayed as mean ± s.d., *n* = 3 biologically independent samples; for **g**–**j**, *n* = 4 biologically independent samples, two-sided Student’s *t* test. For analyses with more than four comparisons, *P < 0.05, **P < 0.01. Source data are provided as a Source Data file.

Fmp30p exhibits high overall sequence and predicted structural similarity to the human phospholipase D enzyme, NAPE-PLD (33% identity, 51% similarity) (Fig. 2a). Both proteins belong to the metallo-β-lactamase superfamily and share the highly conserved HxHxDH motif that contains the metal-binding residues critical for catalysis (Supplementary Fig 2a)^20^. This high similarity underlies the initial claim that Fmp30p is a yeast enzyme with NAPE-specific phospholipase D activity^21^, although this finding has been debated^22,23^. We purified recombinant NAPE-PLD and Fmp30p (Supplementary Fig 2b-d), verified their phosphodiesterase activity against the generic substrate Bis-pNPP (Supplementary Fig 2e), and tested whether they could hydrolyze N-acylphosphatidylethanolamine (NAPE), the native substrate of NAPE-PLD (Supplementary Fig 2f). While NAPE-PLD rapidly depleted 18:1-PE-N-18:1 (18:1 NAPE) to generate 18:1 N-acylethanolamide (18:1 NAE), recombinant Fmp30p had no measurable activity toward this substrate (Fig. 2b, c, Supplementary Fig 2g).

Fmp30p exhibits two critical differences to NAPE-PLD: Fmp30p contains an N-terminal mitochondrial targeting sequence and a single-pass transmembrane helix absent in the human protein (Fig. 2a). Interestingly, only budding yeast retain both these mitochondrial localization and transmembrane domains, suggesting these proteins may have evolved alternative functions (Supplementary Fig 2h). Consistently, neither wild-type nor mitochondrially-localized *NAPE-PLD* rescued the CoQ phenotype of *fmp30*Δ (Fig. 2d). Additionally, an *FMP30* construct with a mutated metal-binding HxHxDH motif (H265N) did not rescue this phenotype (Fig. 2d), implying that Fmp30p’s putative hydrolase activity is critical for its function.

Since the catalytic core and active site of Fmp30p and NAPE-PLD are highly conserved (Supplementary Fig 2i), we reasoned that Fmp30p may possess phospholipase activity toward a different mitochondrial phospholipid substrate. Surprisingly, of all phospholipids assays performed, only those with phosphatidylinositol (PI) yielded phosphatidic acid (PA) (Fig. 2e), a common product of a phospholipase D reaction. Importantly, we observed little-to-no generation of PA with either NAPE-PLD or the catalytically dead Fmp30p H265N mutant using PI as a substrate (Fig. 2f). Given the differences in cellular localization and substrate specificity of NAPE-PLD and Fmp30p, we postulate that these proteins have evolved distinct cellular functions.

To probe the activity of Fmp30p in vivo, we isolated highly purified mitochondria from a wild-type, *fmp30*Δ, and wild-type strain over-expressing *FMP30* driven by a high-expression promotor (GPD) (Supplementary Fig 3a) and performed both targeted and untargeted lipidomic analyses. Consistent with our observations at the whole cell level, deletion of *FMP30* increased the mitochondrial CoQ:PPHB ratio, while *FMP30* overexpression produced the opposite effect (Fig. 2g). Deletion of *FMP30* significantly increased levels of nearly all PI species in purified mitochondria (Fig. 2h). Reciprocally, overexpression of *FMP30* led to a unique loss of PI species (Fig. 2i). Notably, PI is the only phospholipid class that demonstrated such drastic changes in scenarios of both deletion and overexpression of *FMP30* (Supplementary Fig 3b, c). These expression models exhibited the opposite effect on PA abundance, with decreased PA levels in the *FMP30* deletion and increased levels in the overexpression strain (Fig. 2j), supporting a PLD activity. Finally, incubation of recombinant Fmp30p with mitochondria isolated from *fmp30*Δ yeast significantly increased the levels of numerous PA species, with no increase in PA observed with the Fmp30p H265N mutant (Fig. 2k). Collectively, these data point to PI as the native substrate of Fmp30p.

We next tested if the observed Fmp30p phenotypes were consistent across other growth conditions and yeast strains. To accomplish this, we first cultured BY4742 wild-type and *fmp30*Δ cells in standard fermentative media containing glucose, in contrast to the respiratory media primarily used in this study. Under these conditions, cells lacking *FMP30* demonstrated a similar CoQ phenotype (Supplementary Fig 3d-f), albeit with a lower effect size than seen in respiratory media. We next examined the function of Fmp30p in the alternative yeast strain W303. Isolated pure mitochondria from W303 yeast showed similar increases in mitochondrial PI species in an *FMP30* deletion compared to the wild-type counterpart (Supplementary Fig 3g, h), confirming Fmp30p’s role as a mitochondrial PI hydrolase. However, this elevation of mitochondrial PI did not result in an increase in CoQ abundance (Supplementary Fig 3i). Interestingly, wild-type W303 contained over threefold more CoQ than the BY4742 strain and had undetectable levels of PPHB (Supplementary Fig 3j, k). This profile of wild-type W303 yeast is remarkably similar to the BY4742 *fmp30*Δ strain, and it suggests that CoQ headgroup modification may not be the rate-limiting biosynthetic step in the W303 background.

### Mitochondrial PI abundance modulates CoQ biosynthesis

**Fig. 3:**
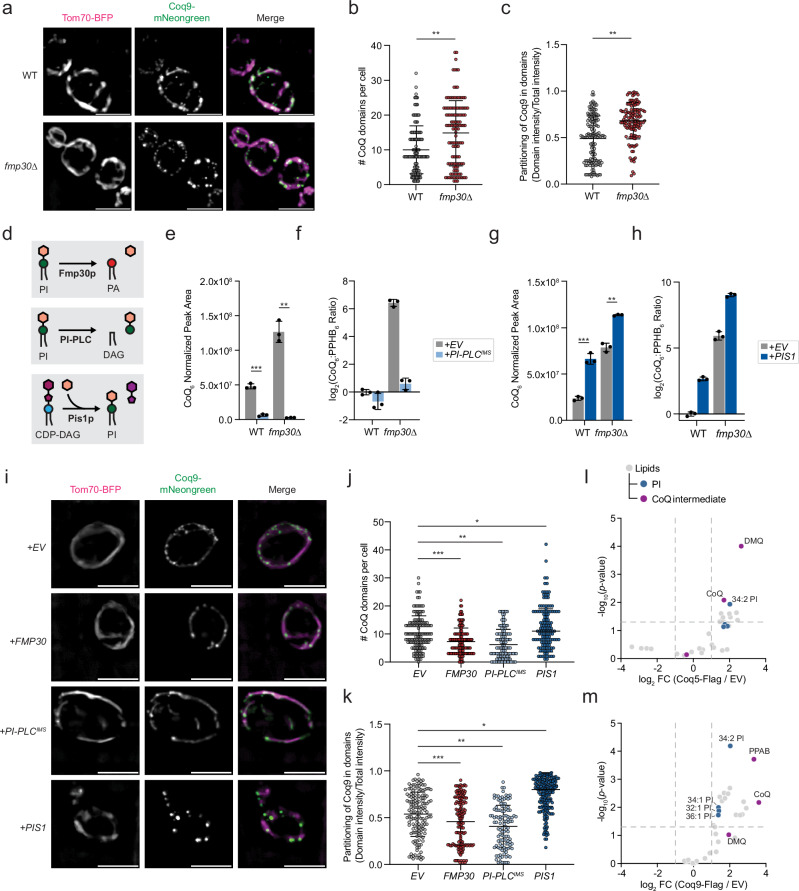
Mitochondrial PI abundance regulates CoQ biosynthesis. **a** Representative max projection z-stack images of WT or *fmp30*Δ cells expressing endogenously-tagged Coq9-mNeonGreen (green) and Tom70-BFP (magenta). Scale bar = 5 μm. Quantification of the number of CoQ domains per cell (**b**) or partitioning of Coq9p into domains as calculated by the fraction of domain intensity over total Coq9p fluorescence intensity (**c**) of the cells imaged in **a** (**b**, **P = 1.71 × 10^−6^; **c**, **P = 1.18 × 10^−10^). **d** Schematic of the reactions catalyzed by Fmp30p, PI-PLC, and Pis1p. Abundance of CoQ_6_ (**e**) and the Log_2_ transformed ratio of CoQ_6_ to PPHB_6_ abundance (normalized to WT) (**f**) of WT and *fmp30*Δ strains expressing an empty vector (*EV*) or an IMS-localized PI-specific phospholipase C (*PI-PLC*^*IMS*^) (**e**, ***P = 5.96 × 10^−5^, **P = 1.50 × 10^−4^). Abundance of CoQ_6_ (**g**) and the Log_2_ transformed ratio of CoQ_6_ to PPHB_6_ abundance (normalized to WT) (**h**) of WT and *fmp30*Δ strains expressing an empty vector (*EV*) or the phosphatidylinositol synthase (*PIS1*) (**g**, ***P = 2.72×10^−4^, **P = 2.12 × 10^−4^). **i** Representative max projection z-stack images of WT cells carrying an empty vector or *FMP30*, *PIS1*, or *PI-PLC*^*IMS*^, expressing endogenously-tagged Coq9-mNeonGreen (green) and Tom70-BFP (magenta). Scale bar = 5 μm. Quantification of the number of CoQ domains per cell (**j**) or partitioning of Coq9p into domains as calculated by the fraction of domain intensity over total Coq9p fluorescence intensity (**k**) of the cells imaged in **I** (**j**, ***P = 2.18 × 10^−6^, **P = 1.35 × 10^−8^, *P = 3.69 × 10^−2^; **k** ***P = 1.85 × 10^−2^, **P = 2.35 × 10^−5^, *P < 1.00 × 10^−15^). Relative lipid abundance versus statistical significance of lipids enriched following mitochondrial isolation and FLAG immunoprecipitation in a WT strain overexpressing *COQ5*-FLAG (**l**) or *COQ9*-FLAG (**m**) compared to WT expressing an empty vector. PI species are highlighted in blue, CoQ intermediates are highlighted in purple. For **e**–**h** and **l**–**m**, data are displayed as mean ± s.d., *n* = 3 biologically independent samples, two-sided Student’s *t* test. For **b, c**, all data points are displayed with the mean ± s.d. indicated, *n* = 141 (for WT) or *n* = 129 (for *fmp30*Δ) cells from three independent experiments, two-sided Welch’s *t*-test. For **j**, **k**, all data points are displayed with the mean ± s.d. indicated, *n* = 149 (for +*EV*), *n* = 146 (for +*FMP30*), *n* = 119 (for +*PI-PLC*^*IMS*^), or *n* = 130 (for +*PIS1*) cells from three independent experiments, Brown-Forsythe and Welch one-way ANOVA tests. Source data are provided as a Source Data file.

As *FMP30* deletion specifically altered mitochondrial phosphatidylinositol levels (Fig. 2h) and increased complex Q abundance (Fig. 1j), we hypothesized that localized increases in PI membrane concentration may modulate complex Q formation in the *fmp30*Δ strain. To examine this, we assessed how *FMP30* deletion affected complex Q domains—large foci of CoQ proteins that often reside near ER contact sites^24,25^. Indeed, *FMP30* deletion increased the number and intensity of CoQ domains and shifted the partitioning of Coq9p-mNeonGreen from diffuse mitochondrial localization (indicative of an inactive complex Q)^24^ into discrete domain foci (Fig. 3a-c, Supplementary Fig 4a).

To further probe this model, we generated additional strains capable of altering mitochondrial PI levels via ectopic expression of PI-modifying enzymes (Fig. 3d). Selectively depleting mitochondrial PI levels by overexpressing an IMS-localized PI-phospholipase C (*PI-PLC*^*IMS*^) from *B. cereus*^26^ markedly disrupted CoQ biosynthesis in both wild-type and *fmp30*Δ cells and reverted the CoQ:PPHB ratio in the *fmp30*Δ back to wild-type levels (Fig. 3e, f, Supplementary Fig 4b). Conversely, overexpression of phosphatidylinositol synthase 1 (*PIS1*) to increase PI levels resulted in the inverse phenotype, further increasing the PI content in *fmp30*Δ cells (Supplementary Fig 4c) and modestly increasing CoQ levels in both wild-type and *fmp30*Δ cells (Fig. 3g, h, Supplementary Fig 4d). Both enzymes were overexpressed to a similar extent as estimated by mass spectrometry (Supplementary Fig 4e, f). Purified mitochondria from cells overexpressing *PIS1* (Supplementary Fig 4g) exhibited similar modest increases in PI levels (Supplementary Fig 4h) and alterations to the CoQ:PPHB ratio were comparable to the whole cell measurements (Supplementary Fig 4i-k). This increase in CoQ was proportional to the increase in the abundance of complex Q proteins in the *PIS1* overexpression strain (Supplementary Fig 4f).

Despite the modest increases in CoQ abundance, there were stark differences in both the number and intensity of CoQ domains detected in *PIS1-*overexpressing cells (Fig. 3i-k, Supplementary Fig 4l), suggesting that even small, localized increases in PI can drastically increase metabolon formation in the absence of a significant increase in protein levels. The inverse relationship between domain formation and the expression of PI-depleting enzymes was observed in cells overexpressing *FMP30* or the *PI-PLC*^*IMS*^ (Fig. 3i-k, Supplementary Fig 4l), in which more Coq9p signal was diffusely mitochondrial rather than localized to discrete CoQ domain puncta.

In addition to elevated mitochondrial PI in the absence of *FMP30*, we detected a slight decrease in total and remodeled species of cardiolipin (CL) in *fmp30*Δ pure mitochondria (Supplementary Fig 4m, n). Therefore, to exclude CL as the driving factor for changes in CoQ biosynthesis, we generated a strain lacking the CL biosynthetic enzyme Tam41p, which exhibited a near complete loss of CL (Supplementary Fig 4o), yet revealed no increase in CoQ (Supplementary Fig 4p-r). We observed no other significant changes in the total levels of other phospholipid classes in the *fmp30*Δ or *PIS1* overexpressing strain (Supplementary Fig 4s-u). Similarly, though overexpression of *PI-PLC*^*IMS*^ drastically altered CL levels, as expected with loss of mitochondrial content (Supplementary Fig 4e), no consistent directional changes in other phospholipid classes were observed (Supplementary Fig 4v). Additionally, we saw no significant differences in phospholipid class abundance in the ER lipidomes from wild-type, *fmp30*Δ, and *PIS1* overexpressing cells (Supplementary Fig 4w-z) and no changes in the expression of protein markers indicating the induction of an ER stress response (Supplementary Fig 4f). Collectively, these experiments strongly support mitochondrial PI as the primary factor driving the observed effects on CoQ domain formation and biosynthetic flux.

**Fig. 4:**
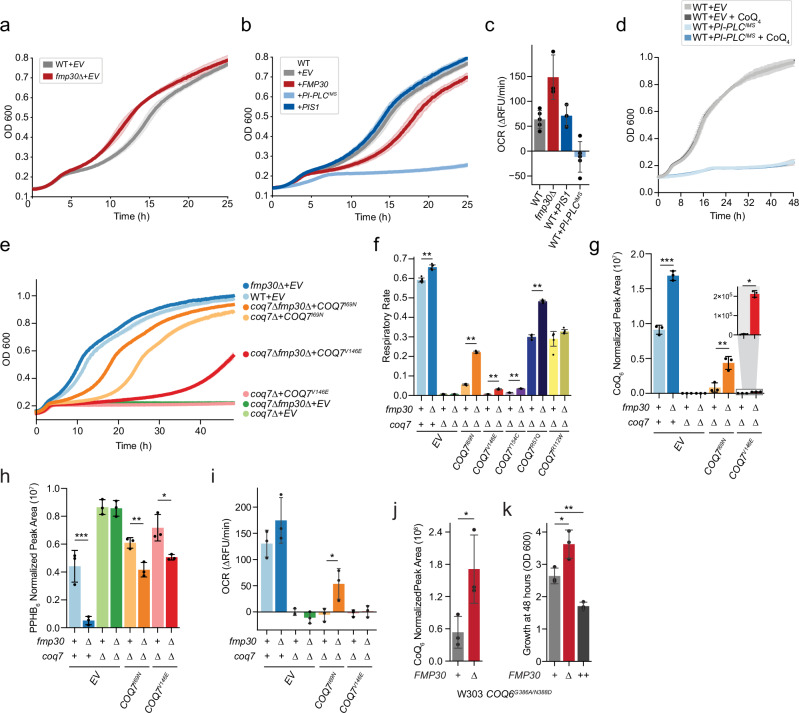
Deletion of *FMP30* partially rescues pathogenic mutations in complex Q genes. Respiratory growth assays of WT and *fmp30*Δ cells expressing an *EV* (**a**) or WT expressing an *EV*, *FMP30*, *PI-PLC*^*IMS*^, or *PIS1* (**b**). WT + *EV* samples are the same in **a** and **b**. **c** Oxygen consumption rate (OCR) of WT or *fmp30*Δ cells expressing the indicated construct, measured as a change in relative fluorescence over a linear period of 45 min. *n* = 5 biologically independent samples for WT and WT + *PI-PLC*^*IMS*^, *n* = 3 biologically independent samples for *fmp30*Δ and WT + *PIS1*. **d** Respiratory growth assay of WT cells harboring an EV or expressing *PI-PLC*^*IMS*^, grown in respiratory media supplemented with 10 μM CoQ_4_ or vehicle. **e** Respiratory growth assays of WT, *coq7*Δ, *fmp30*Δ, or *coq7*Δ*fmp30*Δ cells expressing the indicated mutant *COQ7* construct. **f** Quantification of the respiratory growth rates depicted in **e**. Abundance of CoQ_6_ (**g**) and PPHB_6_ (**h**) in WT, *coq7*Δ, *fmp30*Δ, or *coq7*Δ*fmp30*Δ cells expressing the indicated mutant *COQ7* construct (**g**, ***P = 1.34 × 10^−4^, **P = 6.05 × 10^−3^, *P = 2.97 × 10^−5^; **h**, ***P = 4.63 × 10^−3^, **P = 6.49 × 10^−3^, *P = 1.93 × 10^−2^). **i** Oxygen consumption rate (OCR) of WT, *coq7*Δ, *fmp30*Δ, or *coq7*Δ*fmp30*Δ cells expressing the indicated mutant *COQ7* construct, measured as a change in relative fluorescence over a linear period of 45 minutes (*P = 3.85 × 10^−2^). **j** Abundance of CoQ_6_ in W303 *COQ6*^*G386A/N388D*^ and W303 *COQ6*^*G386A/N388D*^ *fmp30*Δ cells (*P = 4.39 × 10^−2^). **k**, Growth assay of W303 *COQ6*^*G386A/N388D*^, W303 *COQ6*^*G386A/N388D*^ *fmp30*Δ, and W303 *COQ6*^*G386A/N388D*^ overexpressing *FMP30*. Growth was determined after 48 h in respiratory media (*P = 2.73 × 10^−2^, **P = 4.03 × 10^−3^). For **a**–**k**, data are displayed as mean ± s.d., unless otherwise noted *n* = 3 biologically independent samples, two-sided Student’s *t* test. For analyses with more than four comparisons, *P < 0.05, **P < 0.01. Source data are provided as a Source Data file.

**Fig. 5:**
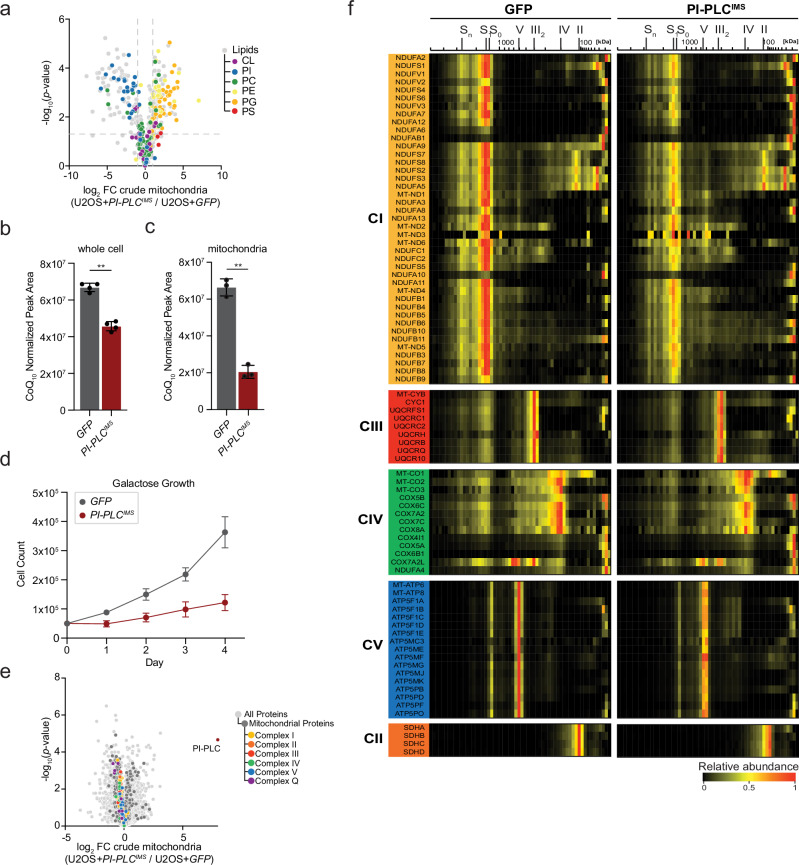
Mitochondrial PI is critical for CoQ biosynthesis in mammalian cells. **a** Relative lipid abundance in mitochondria isolated from U2OS cells expressing the IMS-localized *PI-PLC* compared to U2OS cells expressing *GFP*, versus statistical significance. Points are color-coded according to lipid class. Abundance of CoQ_10_ in whole cells (**b**) or isolated mitochondria (**c**) of U2OS stably expressing *GFP* or *PI-PLC*^*IMS*^ (**b**, **P = 1.69 × 10^−5^; **c**, **P = 1.67 × 10^−4^). **d** Growth assay of U2OS cells expressing GFP or *PI-PLC*^*IMS*^ to measure respiratory growth in galactose-containing media. **e** Relative protein abundances from isolated mitochondria of U2OS cells expressing *PI-PLC*^*IMS*^ compared to U2OS control cells expressing *GFP*, versus statistical significance. Proteins are color-coded according to mitochondrial oxidative phosphorylation (OxPhos) complexes. **f** OxPhos complexes of U2OS cells expressing GFP or *PI-PLC*^*IMS*^ analyzed by complexome profiling. Complexome profiling data are presented as a heatmap, corresponding to subunits of individual OxPhos complexes I–V. Mitochondrial complexes were solubilized with digitonin, separated by blue native polyacrylamide gel electrophoresis, and gel slices were analyzed by quantitative MS. The relative abundance of each protein across all gel slices is represented by the color scale illustrated on the bottom right. For **a** and **e**, *n *= 3 biologically independent samples, two-sided Student’s *t* test. For **b**–**d**, data are displayed as mean ± s.d., *n* = 3 (**b,** **d**) or *n* = 4 (**c**) biologically independent samples, two-sided Student’s *t* test. Source data are provided as a Source Data file.

We next explored how increased mitochondrial PI could lead to changes to complex Q abundance and distribution. Mitochondrial crosslinking AEMS experiments demonstrated that complex Q composition remained unchanged in *fmp30*Δ cells despite the increased abundance, suggesting that new proteins were not being recruited to the complex (Supplementary Fig 1c, d). We instead explored if complex Q may be interacting with PI directly to aid in seeding or stabilizing metabolon formation. To test this, we overexpressed a panel of FLAG-tagged complex Q proteins in yeast, isolated mitochondria, and performed FLAG immunoprecipitation with stringent washing followed by lipidomic analyses. Intriguingly, in addition to the robust enrichment of the expected CoQ intermediates, we detected prominent enrichment of multiple abundant PI species, particularly PI 34:2, with several, but not all, complex Q baits (Fig. 4l, m, Supplementary Fig 4aa-ac). However, a similar enrichment was seen with Ptc7p-FLAG, a matrix phosphatase with multiple reported functions^27–29^ (Supplementary Fig 4a, d). This could suggest that PI has interactions with multiple mitochondrial proteins beyond complex Q, a hypothesis consistent with our complexome profiling experiments below (Fig. 5f). Overall, these findings provide initial clues for how mitochondrial PI could support CoQ biosynthesis and general mitochondrial protein architecture and stability.

### Deletion of *FMP30* partially rescues pathogenic mutations in complex Q genes

To understand the phenotypic consequences of manipulating mitochondrial PI levels and CoQ biosynthesis, we measured respiratory growth rate and oxygen consumption rate (OCR) in strains lacking Fmp30p or expressing PI-modifying enzymes. Deletion of *FMP30* resulted in a slight improvement in respiratory growth and OCR compared to wild-type cells (Fig. 4a, c), consistent with the increase in CoQ content in this strain. While overexpression of *PIS1* did not significantly alter either metric, expression of *PI-PLC*^*IMS*^ led to a dramatic decrease in the respiratory growth rate and OCR (Fig. 4b, c). Interestingly, expression of *PI-PLC*^*IMS*^ also resulted in a marked loss in overall mitochondrial protein content (Supplementary Fig 4e), and its severe respiratory defect could not be rescued by supplementation with CoQ_4_ (Fig. 4d), suggesting that PI may have broader fundamental, yet unexplored, roles in mitochondrial biology.

With the observed increases in CoQ, respiratory growth rate, and OCR, we postulated that perturbation of *FMP30* function may represent a strategy to overcome mitochondrial dysfunction resulting from CoQ deficiencies. To explore this hypothesis, we generated several *COQ7* constructs harboring mutations analogous to mutations found in human patients (R57Q, I69N, R112W, V146E, and Y154C)^30^ and expressed these constructs in *coq7*Δ yeast. Expression of these mutant constructs resulted in respiratory growth defects of varying extents, recapitulating recently published findings^30^, that were subsequently rescued with the deletion of *FMP30* in all but one of the mutant conditions (Fig. 4e, f). Measuring the CoQ abundance (Fig. 4g), PPHB abundance (Fig. 4h), and OCR (Fig. 4i) in a subset of these strains revealed that deficiencies in all metrics mirrored the observed growth deficiencies and were partially rescued upon deletion of *FMP30* (Fig. 4g-i). We observed similar CoQ abundance and respiratory growth rescues upon deletion of *FMP30* in a W303 strain harboring an enzymatically deficient *COQ6* mutation^31^ (Fig. 4j, k). Collectively, these results highlight the potential utility of modulating mitochondrial PI levels as means to augment endogenous CoQ production.

### Mitochondrial PI is critical for CoQ biosynthesis in mammalian cells

We next explored whether the general function of mitochondrial PI in modulating CoQ biosynthesis is conserved in a mammalian context. We first asked if disruption to the function of NAPE-PLD, the Fmp30p mammalian homolog, impacted CoQ levels. To accomplish this, we treated human U2OS cells with the NAPE-PLD inhibitor LEI-401, which has been shown to significantly increase NAPE abundance^32^. Accordingly, treatment with LEI-401 increased NAPE levels in U2OS cells, demonstrating significant inhibition of NAPE-PLD (Supplementary Fig 5a). Despite this inhibition, no changes in the level of CoQ_10_ were observed in these cells (Supplementary Fig 5b), demonstrating that NAPE-PLD does not play a role in CoQ regulation.

Given the divergent substrate preference between NAPE-PLD and Fmp30p (Fig. 2b, f), we manipulated PI levels in human U2OS cells by stably expressing an IMS-localized human PI-PLC construct. Phenocopying the observations in yeast, expression of *PI-PLC*^*IMS*^ in these cells drastically reduced the abundance of mitochondrial PI species (Fig. 5a) and significantly decreased whole-cell and mitochondrial CoQ_10_ levels (Fig. 5b, c). Reduction of mitochondrial PI content also led to the reduced respiratory growth rate when grown in galactose-containing media (Fig. 5d). Importantly, we have not previously observed CoQ abundance defects as a consequence of OxPhos dysfunction in yeast^16^ (Supplementary Fig. 5c) or mammalian cells^10^ (Supplementary Fig. 5d), demonstrating that these phenotypes are specific to a loss in mitochondrial PI and not general respiratory deficiency.

This reduction in respiratory growth rate was to a greater extent than might be expected based on the decreased abundance of CoQ alone, suggesting other potential ramifications stemming from the loss of mitochondrial PI. To examine these and other potential phenotypes, we measured mitochondrial protein abundance using proteomics, as well as mitochondrial complex formation using complexome profiling (Supplementary Fig 5e). While a slight decrease in the steady state abundance of OxPhos subunits was observed with *PI-PLC*^*IMS*^ expression compared to control samples (Fig. 5e), much greater losses were observed in the abundance of fully assembled OxPhos complexes and super-complexes, particularly those containing complex I (Fig. 5f, Supplementary Fig 5f). Combined, these data suggest that PI is critical not just for the formation or stability of complex Q but also for the organization and formation of multiple mitochondrial enzyme complexes.

## Discussion

Here, we identify the uncharacterized mitochondrial protein Fmp30p as a lipid-modifying hydrolase and regulator of CoQ biosynthesis in *S. cerevisiae*. Using genetic manipulation, untargeted lipidomics, and biochemical approaches, we characterize Fmp30p as a mitochondrial phospholipase type D enzyme that hydrolyzes phosphatidylinositol to yield phosphatidic acid, reshaping the composition of the mitochondrial inner membrane. In the absence of *FMP30*-mediated mitochondrial phosphatidylinositol modulation, we observe various factors that support enhanced biosynthetic flux through the CoQ pathway: increased steady state and de novo CoQ abundance, increased complex Q protein content, and increased CoQ domain formation. These CoQ increases are accompanied by loss of the early intermediate PPHB and accumulation of the penultimate intermediate DMQ.

Our work strongly supports a model in which elevated mitochondrial PI in the *fmp30*Δ strain drives an increase in CoQ biosynthesis, especially given the consistent yet opposite effects of *PIS1* and *PI-PLC*^*IMS*^ overexpression on mitochondrial PI content and CoQ biosynthesis. Though previous observations linked *FMP30* to cardiolipin (CL) biology^23^, we show that loss of CL, a critically important phospholipid for mitochondrial function, is not necessary or sufficient to drive the observed changes in CoQ biosynthesis. Finally, we propose that the relationship between mitochondrial PI and CoQ biosynthesis is conserved in mammals, and that PI is critical in regulating additional aspects of mitochondrial function.

How altering PI content within the IMM contributes to complex Q formation remains an open question. In the most straightforward model, complex Q proteins may directly bind to PI within the inner leaflet of the IMM, thus facilitating complex formation and/or promoting stabilization. The potential hydrogen bonding abilities of the inositol headgroup of PI make it an intriguing candidate for such a role, as similar mechanisms are commonly observed with phosphoinositides^33^. Consistent with this model, we enriched multiple abundant PI species when immuno-purifying complex Q proteins; however, proving such a model is technically challenging given the difficulty in verifying protein-lipid interactions using conventional techniques^34^. Newly described methods, such as bioactive lipid probes, may be better suited to unequivocally identify such protein-lipid interactions^35^. Alternatively, PI levels within the inner mitochondrial membrane could change biophysical properties of the membrane, such as curvature or rigidity, that may promote complex formation, as seen with other mitochondrial protein complexes^36^. Once flux through the pathway is established, we and others have demonstrated that the presence of the resulting intermediates is required to the maintain complex Q stability^18,24^. The precise intermediate necessary for stabilization, however, has yet to be determined. The increased abundance of DMQ in the *FMP30* null condition could point to DMQ as the critical intermediate in our system or, alternatively, the stability of complex Q may be driven by the flux of CoQ intermediates through the biosynthetic pathway and not their steady state abundances.

While PI has been found bound within the structures of mitochondrial proteins^37^, its biological role in these interactions has yet to be defined. A meta-analysis examining protein-phosphoinositide interactions has nominated 240 PI-binding proteins in yeast, with 29 localized to mitochondria^38^, though the functional consequences of such interactions are unclear. Recent studies have begun to highlight new roles of downstream phosphoinositides in regulating important mitochondrial processes^39–41^; however, to our knowledge, our findings here represent the first known function of PI in mitochondria, with Fmp30p as the first reported enzyme to specifically modulate mitochondrial PI levels. This is perhaps surprising given that PI exists in a relatively high abundance in the inner mitochondrial membrane of yeast (~16%) and mammals (5–8%)^42^. Interestingly, we found that depletion of mitochondrial PI in both yeast and mammalian systems resulted in a stark respiratory defect accompanied by a loss of mitochondrial protein content and a lack of assembled OxPhos machinery, respectively, that could not be rescued by CoQ supplementation. This hints at a broader and more profound role of mitochondrial PI beyond regulation of CoQ biosynthesis that has yet to be explored. While we demonstrate the impact of PI on CoQ domain formation and biosynthesis, we speculate that this phenomenon could extend to the regulation of additional mitochondrial membrane complexes.

Even though CoQ biology has been an active area of investigation for over 65 years, regulation of CoQ biosynthesis remains poorly characterized. Some evidence suggests CoQ biosynthesis is regulated at the transcriptional, translational, and post-translational levels; however, such mechanisms are often species- and context-specific^1^. Given the critical role of CoQ in maintaining energy homeostasis and the severe human pathologies that arise from its deficiency, it is imperative to define regulatory mechanisms that govern CoQ biosynthesis. Primary and secondary CoQ deficiencies suffer from largely ineffective therapeutic options due to the high hydrophobicity and low bioavailability of CoQ^5^. Alternative options currently being explored involve chemical bypass of dysfunctional steps in the biosynthetic process^43–45^ or manipulation of the pathway by small molecule probes to boost CoQ biosynthesis^46^. Of note, our results in the alternative yeast strain W303 suggest that boosting CoQ levels may be dependent on the rate-limiting steps of its biosynthesis, which should therefore be taken into consideration for the future development of therapeutics.

Our findings demonstrate that mitochondrial PI enhancement is sufficient to adequately boost CoQ production in yeast crippled by patient-related CoQ enzyme mutations, suggesting that mitochondrial lipidome modulation may hold promise as therapeutic strategy for human CoQ deficiency. Furthermore, we establish that the ability of mitochondrial PI to regulate CoQ biosynthesis is conserved, at least in part, in a mammalian context. Identification of a human Fmp30p functional complement could provide an important handle for manipulating CoQ levels in a disease-relevant context. The identification and characterization of potent specific inhibitors of NAPE-PLD^47^ suggest that a mammalian ortholog of Fmp30p could represent a druggable target, thereby exposing a therapeutic avenue to manipulate CoQ biosynthesis in vivo. Collectively, our findings nominate a previously uncharacterized mitochondrial protein, Fmp30p, as a negative regulator of CoQ biosynthesis. Further efforts to understand the coordination of phosphatidylinositol metabolism with CoQ biosynthesis, especially in a mammalian context, may enable therapeutic manipulation of this pathway.

## Methods

### Yeast strain generation and culture conditions

The *S. cerevisiae* haploid strain BY4742 (MATa his3 leu2 lys2 ura3) and W303 (MATa leu2 trp1 can1 ura3 ade2 his3) were used and cultured under standard laboratory conditions. Single (*gene*∆) and double (*gene*∆*gene*∆) deletion strains were generated using PCR-based homologous recombination, where open reading frames were replaced with the KanMX6, Leu2 or His3MX6 cassettes, transformed using standard heat shock conditions and confirmed via PCR genotyping^48^. For complex Q crosslinking immunoprecipitation mass spectrometry experiments, the C-terminal endogenous tag on Coq9 was generated using PCR-based homologous recombination with the mNeonGreen:NatMX6 cassette. For CoQ domain imaging strains containing Coq9-nNeonGreen, the mitochondrial marker Tom70 was C-terminally tagged using the BFP:Leu2 cassette. For mitochondrial morphology imaging, Tom70 was C-terminally tagged using the mNeonGreen:NatMX6 cassette. For ER rapid immunoprecipitation experiments, Sec63 was C-terminally tagged using the mNeonGreen:NatMX6 cassette

For all whole cell lipidomic and proteomic analyses, strains from glycerol stocks were first struck out YPD plates consisting of 1% (w/v) yeast extract (“Y”) (Research Products International), 2% (w/v) peptone (“P”) (Research Products International), 2% (w/v) dextrose (“D”) (Fisher) and 2% (w/v) agar (Sigma-Aldrich) and allowed to grow for 48 h at 30 °C. Starter cultures were inoculated from individual colonies in 3 mL YPD media and incubated for 24 h (30 °C, 230 rpm). Cell density was measured at OD_600_ and 1.25 × 10^6^ cells from each starter culture were used to inoculate 50 mL of respiratory YPG media (1% (w/v) Y, 2% (w/v) P, 0.1% D and 3% glycerol (“G”)) in a sterile 250 mL Erlenmeyer flask. Samples were incubated (30 °C, 230 rpm) and 1 × 10^8^ cells were collected at 24 h, a timepoint that corresponds to early respiratory growth. The samples were collected by centrifugation (4000 × *g*, 5 min, r.t.). The supernatant was removed and the cells were washed with 1 mL of sterile water. Cells were pelleted again (12,000 × *g*, 1 min, r.t.) and the supernatant was removed. Cell pellets were snap frozen in liquid nitrogen (LN_2_) and stored at −80 °C until analysis.

### Plasmids

Yeast *FMP30*, human *NAPE-PLD*, and human *NAPE-PLD* fused with the mitochondrial targeting sequence of yeast *FMP30* were cloned into a single-copy plasmid (p416) under the control of the GPD promoter. C-terminally FLAG-tagged constructs (yeast *COQ3*, *COQ4*, *COQ5*, *COQ6*, *COQ9*, *PTC7*) were also cloned into the p416-GPD vector. Yeast *FMP30, PIS1*, or *COQ7* were cloned into an alternative p416-GPD plasmid in which the *URA3* gene was swapped for the KanMX6 cassette. Point mutants were generated by standard site-directed mutagenesis. To target protein localization to the mitochondrial intermembrane space, the *B. cereus* phosphatidylinositol-specific phospholipase C (PI-PLC, Genbank accession no. AAA22665.1, residues 32-328, mutations W47A, W242A, R163A)^26^ was fused C-terminally to the mitochondrial targeting sequence and transmembrane domain of *FMP30* (residues 1-94) [*PI-PLC*^*IMS*^]. Constructs were transformed into WT, *fmp30*Δ, *coq7*Δ, and *fmp30*Δ*coq7*Δ yeast using the LiAc/SS carrier DNA/PEG method^49^. Strains harboring the p416-GPD URA3 vectors were grown in SC Ura (2% glucose, w/v) or SC Ura^−^ (0.1% glucose, 3% glycerol, w/v) as detailed above. Strains harboring the vectors containing the KanMX6 cassette were cultured as above in rich media supplemented with 200 μg/mL G418 (GoldBio).

### Mammalian cell culture

U2OS wild-type cells were cultured in Dulbecco’s Modified Eagle Medium (DMEM, Thermo) with 10% heat-inactivated FBS (Biotechne) and 1x penicillin-streptomycin (Thermo) at 37 °C and 5% CO_2_. For galactose growth assays, 5 × 10^4^ cells per cell line were seeded in triplicate per well of a 6-well plate and incubated overnight to allow cells to adhere to the plate. Cells were washed with 1x Dulbecco’s phosphate-buffered saline (DPBS) and medium was replaced with glucose-free DMEM (Thermo) supplemented with 25 mM galactose, 10% dialyzed FBS (Biotechne), and 1× penicillin–streptomycin. Cells were collected at the indicated timepoint and cell counts were acquired with the TC20 Automated Cell Counter (Bio-Rad). For NAPE-PLD inhibitor treatments, 7.5 × 10^5^ cells were seeded in triplicate per condition in 10-cm plates and incubated overnight to allow cells to adhere to the plate. Cells were treated for 24 h with 2 μm LEI-401 (MedChem Express) or DMSO as vehicle control. Cell pellets (1.5 × 10^6^ cells) were harvested, snap frozen in liquid nitrogen (LN_2_), and stored at −80 °C until analysis.

### Generation of stable cell lines

Constructs of interest were cloned into pLVX-AcGFP1-N1 (Takara 632154) lentiviral vector under the CMV promoter. The *B. cereus* PI-PLC construct fused to the mitochondrial targeting sequence and transmembrane domain of human SCO2 (residues 1-99) was amplified via PCR with XbaI and XmaI restriction sites, digested, and ligated into the pre-digested pLVX-AcGFP1-N1 vector. Lentiviral particles were produced using the Lenti-X 293 T system (Takara 632180) with the pCMV-VSV-G (Addgene), pMDLg/pRRE (Addgene), and pRS-Rev (Addgene) packaging plasmids. Viral media was collected 48 h post-transfection and processed with a Lenti-X concentrator (Takara 631231), then centrifuged (600 × g, 4 h, 4 °C) before aliquoting, flash-freezing, and storing at −80 °C. To transduce cells, U2OS WT cells were seeded in 6-well plates and incubated overnight. Media was supplemented with 0.5 µg/mL polybrene (Sigma) and 20 µL of lentivirus was added to each well. Cells were incubated for 48 h, then the media was exchanged for DMEM with 10% FBS and 0.5 µg/mL puromycin (Thermo) for 6–8 days with passaging throughout. For proteomic and lipidomic analysis, 6 × 10^5^ cells were collected at 8 days post-transduction, snap-frozen in LN_2_ and stored at −80 °C until analysis.

### Mammalian mitochondrial isolation

Mitochondria were isolated from lentiviral-transduced U2OS cell lines by differential centrifugation^50^. In brief, cells were collected from 9 15-cm plates per replicate and washed with ice-cold PBS. The cell pellet was resuspended in 5 mL of isolation buffer (10 mM MOPS-KOH, pH 7.2, 1 mM EDTA, 250 mM sucrose, 0.5% fatty acid-free BSA, 1x cOmplete EDTA-free Protease Inhibitor Cocktail (Roche)). Cells were lysed using 35 strokes of a tight-fitting glass-Teflon homogenizer. Cellular debris in the lysate was pelleted by centrifugation (600 × *g*, 10 min, 4 °C). The supernatant was transferred to fresh tubes and crude mitochondria were pelleted by centrifugation (12,000 × *g*, 10 min, 4 °C). Mitochondrial pellets were finally washed with isolation buffer without BSA. Total mitochondrial protein content was measured using the Pierce BCA Protein Assay Kit (Thermo) before mitochondria were aliquoted, snap frozen in LN_2_ and stored at −80 °C for further use.

### Complexome profiling

Sample preparation and blue-native polyacrylamide gel electrophoresis (BN-PAGE) were performed as described^51^. In brief, 200 μg aliquots of crude mitochondria were resuspended in solubilization buffer (50 mM imidazole, 500 mM 6-hexaminocaproic acid, 1 mM EDTA) containing 6 g/g digitonin (detergent/protein ratio), incubated for 20 min on ice, then centrifuged (20,000 × *g*, 20 min, 4 °C) to remove insoluble material. Then, 30 μg protein, as determined by BCA assay (Thermo), was combined with 5% glycerol and G-250 Coomassie brilliant blue to a final detergent/dye ratio of 8 g/g and separated on a 4–13% native PAGE (dimension 14 × 14 cm) with the NativeMark protein standard (Thermo). Complexome profiling was performed as described^52^ with some modifications. The gel was stained with Coomassie blue, cut into 60 equal fractions, and transferred to 96-well AcroPrep filter plates (Cytiva). The gel fractions were destained in 50% acetonitrile in 50 mM ammonium bicarbonate, then reduced and alkylated in 25 mM ammonium bicarbonate containing 10 mM TCEP and 40 mM 2-chloroacetemide (15 min, r.t.). The gel slices were dehydrated with acetonitrile, then swelled with 25 mM ammonium bicarbonate containing 16 ng/μL trypsin (Promega) and allowed to digest (overnight, r.t.). The peptides were collected, acidified with 10% trifluoroacetic acid to a pH < 3, and desalted using the EasyPep™ MS Sample Prep Kits Peptide Clean-up Plates (Thermo Scientific). The peptides were dried in a SpeedVac (Thermo Scientific) and stored at −80 °C until analysis.

Peptides were resuspended in 0.2% formic acid and an equal volume of sample was injected for each run. LC separation was performed using the Thermo Vanquish Neo UHPLC system. A 5.5 cm High Throughput μPAC^TM^ Neo UHPLC C18 column (Thermo Scientific) was used with a 12.8 min gradient using mobile phase A consisting of 0.1% formic acid in H_2_O, and mobile phase B consisting of 0.1% formic acid in ACN:H_2_O (80:20, v/v).

An EASY-Spray source was used, and the temperature was set at 50 °C. Each sample was first held at 1.2% B with a 1.5 μL/min flow rate and increased to 10% B over 0.1. The gradient was then increased to 28% B over 8 min, to 56% B over 3.6 min, and to 99% B over 0.12 min. The flow was held at 99% B for 0.98 min for column washing. An Acclaim PepMap C18 HPLC trap column (20 mm × 75 µm, 3 µm) was used for sample loading. MS detection was performed with a Thermo Astral mass spectrometer in positive mode. The source voltage was 1.9 kV, ion transfer tube temperature was set to 280 °C, and the RF lens was at 40%. Full MS spectra were acquired from m/z 380 to 980 at the Orbitrap resolution of 240,000, with the normalized AGC target of 300% (3E6) and a maximum injection time of 5 ms. Data-independent acquisition (DIA) was performed with the Astral analyzer with 300 isolation windows of 2-Th scanning from 380 to 980 m/z. The isolated ions were fragmented using HCD with 26% Normalized Collision Energy. Other settings for DIA include an RF lens of 40%, normalize ACG target of 500% (5E4), and a maximum injection time of 3 ms.

#### Data analysis

Raw files were analyzed by the CHIMERYS search algorithm with INFERYS Rescoring incorporated in Proteome Discoverer v.3.2.0.450 software against human databases downloaded from Uniprot. MS2 Apex (quan in all files) was used as the quantification method for the searches. Each individual protein abundance was normalized to the maximum abundance across all gel slices.

### Respiratory growth assays

Starter cultures (YPD, 3 mL) were inoculated with individual colonies and incubated overnight (30 °C, 230 rpm, 14–16 h). For plate reader-based assays, cells were pelleted and resuspended in respiratory media (YPG) at a density of 5 × 10^6^ cells/mL. Then, 100 μL of the resuspended cells were transferred to a sterile 96-well round-bottom plate (Thermo) with a Breathe-Easy cover seal (Diversified Biotech). Cultures were incubated (30 °C, 1140 rpm) in an Epoch2 plate reader (BioTek) with OD_600_ measured every 10 min. When indicated, wells were supplemented with 10 μM CoQ_4_ or vehicle (isopropanol).

### OCR measurements

The oxygen consumption rate (OCR) was measured using the MitoXpress Xtra Oxygen Consumption Assay kit (Agilent), according to the manufacturer’s protocols. In brief, yeast was cultured as described above. Then, 1 × 10^6^ cells were transferred into a clear, 96-well plate (Thermo) and the volume was adjusted to 100 μl. Next, 10 μl of MitoXpress Xtra probe was added to each well and the liquid was topped with four drops of mineral oil. Changes in fluorescence intensity were measured using a Cytation3 plate reader (BioTek, 30 °C, 1140 rpm, excitation: 380 nm and emission: 650 nm) over a 45 min period. The OCR was calculated using Gen5 v3.02.2 software (BioTek) measuring the ∆relative fluorescent units (RFU) over the linear time frame for each sample.

### CoQ domain imaging

#### Fluorescence microscopy

Cells were grown as detailed above. Upon reaching mid-log phase, 1 mL of cells was collected by centrifugation (4000 ×*g*, 1 min) and resuspended in glucose-free media at approximately one one-hundredth of the original volume. All images were taken as z-stacks using a Nikon Spinning Disk equipped with NIS-Elements software (v. 5.21.00). Images were acquired with a 63× oil objective NA = 1.4 at room temperature.

#### Image analysis

Quantification and analysis of CoQ domains were performed in Fiji/ImageJ (v. 1.54p). For quantification of domain number, max projections were generated from Coq9-mNeongreen z-stacks. Max projections were background subtracted by generating a blurred image using the “*Gaussian blur”* function in ImageJ with a sigma = 8.00. Blurred images were subtracted from the max projection using the “*Image calculator”* function. Background-subtracted projections were segmented using “*Adjust threshold”*. Particles were auto-detected using the “*Analyze particles”* function with a size range of 0.05–2.0 µm^2^ and circularity range of 0.5–1.0. For each image, the number of cells was counted manually. For quantification of fluorescence intensity, masks were generated from Tom70-BFP images, as described above for the Coq9-mNeongreen images. For the Tom70-BFP signal, the “*Analyze particles”* parameters were adjusted to particle size = 0.2–8.0 µm^2^ and circularity = 0.1–1.0. Total fluorescence was quantified by overlaying the Tom70-BFP mask on average intensity projections of the Coq9-mNeongreen signal. To quantify fluorescence intensity outside of the domains, the Coq9-mNeongreen particle mask was subtracted from the Coq9-mNeongreen average intensity projections, and the Tom70-BFP mask was used to quantify remaining fluorescence. Mitochondrial morphology was quantified from Tom70-mNeongreen cells using MitoS and MitoA in the MitoSegNet software^53^. A previously described deep learning model for yeast mitochondria was used for segmentation^54^. For each quantification, *n* ≥ 100 cells across three independent experiments.

### TEM

Yeast was grown in respiratory media, as described above. Preparation of samples for TEM was performed as previously described^55^. Briefly, 1.0 × 10^8^ cells were collected by centrifugation and washed with 0.1 M sodium cacodylate buffer (pH 7.2) containing 1 mM CaCl_2_. The cacodylate buffer was removed and replaced with 1.5% glutaraldehyde diluted in cacodylate buffer. Cells were fixed for 2 h at 4 °C, followed by two washes with cacodylate buffer. The fixed yeast cells were embedded in a 2.5% solution of agarose (Ultra-low Gelling Temperature) at 40 °C. The solidified agarose block was cut into small pieces. After washing with cacodylate buffer, the cell wall was enzymatically digested with zymolyase. Samples were washed again and fixed in 0.5% osmium tetroxide and 0.8% potassium ferrocyanide for 35 min at 4 °C. Samples were subsequently incubated with 1% uranyl acetate overnight on ice. After washing, the samples were dehydrated in ascending ethanol concentrations by using a graduated series of ethanol: 30%, 50%, 70%, 80%, 90%, 95%, and 100% ethanol. Each ethanol step was performed for 8 min at 4 °C followed by two steps of propylene oxide dehydration. Samples were infiltrated and embedded in Epon 812. After polymerization, the sample block was cut into ultrathin sections approximately 70 nm in thickness, and the sections were post-stained with uranyl acetate and lead. TEM images were acquired using a Tecnai 12 fitted with a Gatan OneView camera.

### Yeast mitochondrial isolation

Starter cultures (3 mL, YPD) were inoculated with individual colonies and incubated (30 °C, 230 r.p.m., 14–16 h), then 1 × 10^8^ cells were diluted into 2 L of YPG in a 5 L Erlenmeyer flask and incubated (30 °C, 230 r.p.m.) for 24–26 h to a final OD ≈ 3. For the overexpression studies, WT or *fmp30*Δ strains were transformed with a p416-GPD empty vector, p416-GPD-*FMP30*, or p416-GPD-*PIS1* harboring a KanMX resistance cassette in place of the *URA3* gene. In these cases, the media was supplemented with 200 μg/mL G418 to maintain selection. For immunoprecipitation lipidomics experiments, BY4742 WT was transformed with p416-GPD-*GeneX*-FLAG constructs. In these cases, SC Ura^−^ 2%D or SC Ura^−^ 0.1%D, 3%G was used for growth to maintain selection. Mitochondria were isolated as previously described^56^. Cells were collected by centrifugation (3000 × *g*, 5 min, r.t.), washed with water, and centrifuged again (3000 × *g*, 5 min, r.t.). The wet pellet weight of the cells was determined. Cells were resuspended in 2 mL/g dithiothreitol buffer (100 mM Tris-H_2_SO_4_, 10 mM dithiothreitol, pH 9.4) and shaken slowly (30 °C, 80 r.p.m., 20 min). Cells were pelleted, washed once with 7 mL/g Zymolyase buffer (1.2 M sorbitol, 20 mM potassium phosphate, pH 7.4), and resuspended in 7 mL/g Zymolyase buffer with 3 mg/g Zymolyase20T (Fisher) to generate spheroplasts. The yeast was shaken slowly (30 °C, 80 r.p.m.) for 40 min before being pelleted and washed with 7 mL/g Zymolyase buffer. Pellets were resuspended in 6 mL/g ice-cold homogenization buffer (0.6 M sorbitol, 10 mM Tris-HCl, 1 mM phenylmethylsulfonyl fluoride (PMSF), 0.2% (wt/vol) fatty-acid-free bovine serum albumin (BSA), pH 7.4). Spheroplasts were homogenized using 20 strokes of a tight-fitting glass-Teflon homogenizer and diluted two-fold with homogenization buffer. The homogenate was centrifuged (1500 × *g*, 5 min, 4 °C) to pellet cell debris and nuclei. The supernatant was centrifuged (4000 × *g*, 5 min, 4 °C) to pellet additional debris. Crude mitochondria were isolated by centrifuging the supernatant (12,000 × *g*, 15 min, 4 °C) and resuspended in SEM (250 mM sucrose, 1 mM EDTA, 10 mM MOPS-KOH, pH 7.2). For isolation of pure mitochondria, crude mitochondrial fractions in SEM were incubated with 6.7 μg trypsin (sequencing grade, Promega) and rotated end-over-end overnight (16 h, 4 °C) to disrupt proteinaceous organelle contact tethers^57^. On the following day, digested samples were pelleted by centrifugation (15,000 × *g*, 7 min, 4 °C). Pelleted material was resuspended in 900 μL SEM buffer containing 1 mM PMSF to deactivate trypsin. Resuspended material was pelleted (15,000 × *g*, 7 min, 4 °C) and this was repeated once more. Pelleted crude mitochondria were resuspended in 200 μL SEM + PMSF and then added to a freshly prepared sucrose gradient (bottom to top: 1.5 mL 60% sucrose, 4 mL 32% sucrose, 1.6 mL 23% sucrose, and 1.4 mL 15% sucrose) for separation by ultracentrifugation (134,000 × *g*, 1 h, 4 °C). Enriched mitochondrial samples were recovered at the 32−60% interface and diluted with 30 mL SEM. Mitochondria were pelleted (15,000 × *g*, 10 min, 4 °C) and resuspended in fresh SEM. Mitochondrial protein content was quantified by a BCA protein assay (Thermo). Pure mitochondrial samples were snap-frozen as 25–75 μg aliquots for lipidomic analysis.

### Yeast rapid immunopurification of ER membranes

ER membranes were immunopurified from yeast as previously described^58^ with some modifications. Starter cultures (3 mL, YPD) of BY4742 strains expressing endogenously tagged Sec63-mNeonGreen and the indicated plasmids were inoculated with individual colonies and incubated (30 °C, 230 r.p.m., 14–16 h), then 1.25 × 10^6^ cells from each starter culture were used to inoculate 50 mL of YPG media 250 mL flask. After 24 h, 2.5 × 10^8^ cells were pelleted (1500 × *g*, 5 min, 20 °C) and washed in 0.5 mL KPBS buffer (136 mM KCl and 10 mM KPO_4_, pH 7.25). The cell pellets were resuspended in 300 μL lysis buffer (KPBS with 1 mM PMSF and 1X Roche protease COmplete inhibitor tablet) with glass beads (100 μL, 0.5 mm; BioSpec) and the samples were vortexed using a Vortex Genie for 10 min (3000 rpm, 4 °C) to lyse the cells. The samples were centrifuged (500 × *g*, 3 min, 4 °C) to pellet cell debris and the supernatant was transferred to fresh tubes. An additional 600 μL of lysis buffer was added to the beads, vortexed, and centrifuged (500 × *g*, 4 °C). The supernatant was combined with the previous supernatant, and 100 μL was set aside for whole the cell lipid measurement. The remaining supernatant was added to 30 μL of magnetic mNeonGreen-Trap agarose beads (Chromotek, catalog no. nta-20) and incubated (rotating, 1 h, 4 °C). The beads were washed 3X with lysis buffer (1 mL, 5 min, 4 °C) then the supernatant was removed and the dry beads were subjected to MTBE-based lipid extraction and untargeted LC/MS-MS lipidomics as described below.

### Yeast immunoprecipitation-lipidomics

Crude mitochondria were isolated from yeast (BY4742 WT expressing FLAG-tagged constructs) as described above. Mitochondria (1 mg) were solubilized with 50 mM imidazole, 500 mM 6-hexaminocaproic acid, 1 mM EDTA and 1 g/g digitonin. Solubilized mitochondria were added to anti-FLAG M2 magnetic beads (Sigma M8823) equilibrated with wash buffer (10 mM Tris pH 8.0, 140 mM NaCl, 1 mM EDTA) and incubated (4 °C, 2.5 h, end-over-end). The beads were then washed 5× in lysis buffer containing 0.4% digitonin, then 2× in lysis buffer without digitonin. Protein was eluted into 187.5 μL of elution buffer (10 mM Tris pH 8.0, 140 mM NaCl, 1 mM EDTA, 0.2 mg/mL FLAG peptide, Sigma F3290) with agitation (1 h, 25 °C). The eluate was subjected to MTBE-based lipid extraction and untargeted LC/MS-MS lipidomics as described below.

### Crosslinking affinity enrichment mass spectrometry

Crude mitochondria were isolated from yeast (BY4742 WT or *fmp30*Δ with endogenously tagged Coq9-mNeonGreen) as described above and subjected to chemical crosslinking (1 mg mitochondria, 0.5 mM DSSO (Thermo, catalog no. A33433), 1 h, r.t.). Crosslinking was quenched with 100 mM Tris pH 8.0 followed by centrifugation (15,000 × *g*, 5 min, 4 °C). The mitochondria were then solubilized with 50 mM imidazole, 500 mM 6-hexaminocaproic acid, 1 mM EDTA and 1 g/g digitonin. The bait protein and crosslinked interactors were then enriched by mNeonGreen immunoprecipitation (IP) using magnetic mNeonGreen-Trap agarose beads (Chromotek, catalog no. nta-20), washed and subjected to on-bead tryptic digest. The on-bead crosslinked proteins were denatured with 2 M urea in 200 mM Tris pH 8.0, then reduced with 5 mM DTT (30 min, 56 °C) and alkylated with 15 mM iodoacetamide (30 min, r.t., in the dark). The proteins on-bead were digested (overnight, 37 °C) with 1 μg trypsin (Promega, catalog no. V5113). The digested supernatant was acidified with 10% TFA to a pH of 2 and desalted with 10 mg StrataX solid-phase extraction columns (Phenomenex), then dried under vacuum using a SpeedVac (Thermo Scientific) and stored at −80 °C until MS analysis.

Samples were resuspended in 0.2% formic acid and subjected to LC–MS analysis. LC separation was performed using the Thermo Ultimate 3000 RSLCnano system. A 15 cm EASY-Spray PepMap RSLC C18 column (150 mm × 75 μm, 3 μm) was used at 300 nL/min flow rate with a 90 min gradient using mobile phase A consisting of 0.1% formic acid in H_2_O, and mobile phase B consisting of 0.1% formic acid in ACN/H_2_O (80/20, v/v). An EASY-Spray source was used and the temperature was 35 °C. Each sample run was held at 4.0% B for 5 min and increased to 50% B over 65 min, followed by 8 min at 95% B and back to 4% B for equilibration for 10 min. An Acclaim PepMap C18 HPLC trap column (20 mm × 75 μm, 3 μm) was used for sample loading. MS detection was performed with a Thermo Exploris 240 Orbitrap mass spectrometer in positive mode. The source voltage was set to 1.8 kV, ion transfer tube temperature was set to 275 °C, RF lens was at 70%. Full MS spectra were acquired from m/z 350 to 1400 at the Orbitrap resolution of 60,000, with the normalized automatic gain control target of 300% (3 × 10^6^). Data-dependent acquisition (DDA) was performed for the top 20 precursor ions with the charge state of 2–6 and an isolated width of 2. Intensity threshold was 5 × 10^3^. Dynamic exclusion was 30 s with the exclusion of isotopes. Other settings for DDA include Orbitrap resolution of 15,000 and high-energy collision-induced dissociation energy of 30%.

Raw files were analyzed by SequestHT search engine incorporated in Proteome Discoverer v.2.5.0.400 software against yeast databases downloaded from Uniprot. Label-free quantification was enabled in the searches. The resulting data were analyzed by Perseus v.1.6.15.0 software^59^.

### LC-MS/MS proteomics

#### Yeast growth

Yeast cultures were grown as described previously for respiration, then 1 × 10^8^ cells were collected, snap-frozen in LN_2_, and stored at −80 °C.

#### Lysis and digestion

Yeast pellets were removed from −80 °C and resuspended in lysis buffer (6 M guanidine hydrochloride, 100 mM Tris). The samples were then boiled (100 °C, 5 min) and subjected to probe sonication (2 rounds of 10 s on, 20 s off, 25% amplitude). Methanol was added to each sample to 90% concentration, and the samples were centrifuged (9000 × *g*, 30 min) to precipitate proteins. After precipitation, the supernatant was discarded from each sample, and the protein pellets were allowed to air-dry. The dried pellets were resuspended in digestion solution (8 M urea, 10 mM TCEP, 40 mM CAA, 100 mM Tris pH 8.0), and the samples were sonicated in the bath sonicator to facilitate re-solubilization. Trypsin (Promega, catalog no. V5113) was added to each sample at a 50:1 protein/enzyme ratio before they were incubated at 37 °C overnight. The peptides were dried under vacuum using a SpeedVac and resuspended in 0.1% TFA. High pH fractionation was performed according to the manufacturer’s protocol (Pierce High pH Reversed-Phase Peptide Fractionation Kit, Thermo Scientific). Eight fractions were collected and dried under vacuum (Thermo Scientific).

#### Mammalian mitochondrial digestion

Following MTBE-based lipidomic extractions of mammalian isolated mitochondrial samples, the protein content remaining in the aqueous phase was precipitated by centrifugation (1000 × *g*, 3 min), then the protein pellets were washed with ACN. The supernatant was removed and the protein pellets were allowed to dry. The proteins were resuspended in lysis buffer (8 M urea in 100 mM Tris pH 8.0) and subjected to to probe sonication (2 rounds of 10 s on, 20 s off, 25% amplitude). The samples were reduced by incubating with 100 mM DTT (30 min, 50 °C), then alkylated with 300 mM iodoacetamide (30 min, r.t.), prior to dilution by 4× with 100 mM Tris pH 8.0. Trypsin (2 μg, Promega, catalog no. V5113) was added to each sample before they were incubated at 37 °C overnight. The samples were acidified with 10% TFA then desalted with Pierce Desalting Columns (Thermo Scientific) according to the manufacturer’s protocol. The samples were dried under vacuum using a SpeedVac (Thermo Scientific) and stored at −80 °C until MS analysis.

#### LC-MS/MS proteomics data acquisition for fractionated samples

Peptides were resuspended in 0.2% formic acid, and the concentration of each sample was determined using a NanoDrop One spectrophotometer (Thermo Scientific). LC separation was performed using the Thermo Ultimate 3000 RSLC nano system. A 15 cm EASY-Spray PepMap RSLC C18 column (Thermo, 150 mm × 75 μm, 3 μm) was used at 300 nL/min flow rate with an Acclaim PepMap C18 HPLC trap column (Thermo, 20 mm × 75 μm, 3 μm) for sample loading. For each sample run, the temperature was held at 35 °C for a 120 min gradient that consisted of 4% B for 5 min and increased to 30% B over 100 min, followed by 5 min at 99% B and back to 4% B for equilibration for 10 min. Mobile phase A consisted of 0.1% FA in water, and mobile phase B consisted of 0.1% FA in 80% (v/v) ACN and 20% (v/v) water. MS detection was performed with a Thermo Exploris 240 Orbitrap mass spectrometer with an EASY-Spray source operating in positive mode. The source voltage was 1.8 kV, the ion transfer tube temperature was set to 275 °C and the RF lens at 70%. Full MS spectra were acquired from m/z 350 to 1400 at the Orbitrap resolution of 60,000, with a normalized AGC target of 300% (3 × 10^6^). Data-dependent acquisition was performed with a 3 s duty cycle with a charge state of 2–6, an isolation window width of 2 and an intensity threshold of 5 × 10^3^. Dynamic exclusion was 20 s with the exclusion of isotopes. Other settings for data-dependent acquisition were an Orbitrap resolution of 15,000 and higher energy collisional dissociation energy of 30%.

#### Data analysis

Raw files were analyzed using the SequestHT Search Engine incorporated in Proteome Discoverer v.2.5.0.400 software against yeast databases downloaded from Uniprot. Label-free quantification was enabled in the searches.

#### LC-MS/MS proteomics data acquisition for whole cell samples

Peptides were desalted with Pierce peptide desalting spin columns (Thermo Fisher Scientific) and dried under vacuum (Thermo Fisher Scientific). Peptides were resuspended in 0.2% formic acid, and the concentration of each sample was determined using the Pierce Quantitative Fluorescent Peptide Assay (Thermo Scientific). LC separation was performed using the Thermo Vanquish Neo UHPLC system. A 15 cm EASY-Spray PepMap Neo UHPLC C18 column (150 mm × 75 µm, 3 µm, Thermo Scientific) was used with a 24 min gradient using mobile phase A consisting of 0.1% formic acid in H_2_O, and mobile phase B consisting of 0.1% formic acid in ACN:H_2_O (80:20, v/v). An EASY-Spray source was used and the temperature was set at 35 °C. Each sample was first held at 4% B for 0.5 min with an 800 nL/min flow rate, then the flow rate was dropped to 500 nL/min and increased to 8% B over 0.1 min and held there for 0.3 min. The gradient was then increased to 22.5% B over 13.9 min, to 35% B over 6.9 min, and 55% B over 0.4 min. The flow rate was adjusted to 800 nL/min and increased to 99% B over 0.5 min, followed by 2.3 min at 99% for column washing. An Acclaim PepMap C18 HPLC trap column (20 mm × 75 µm, 3 µm) was used for sample loading.

MS detection was performed with a Thermo Astral mass spectrometer in positive mode. The source voltage was 1.9 kV, ion transfer tube temperature was set to 280 °C, and RF lens was at 40%. Full MS spectra were acquired from m/z 380 to 980 at the Orbitrap resolution of 240,000, with the normalized AGC target of 500% (5E6) and a maximum injection time of 3 ms. Data-independent acquisition (DIA) was performed with the Astral analyzer with 300 isolation windows of 2-Th scanning from 380 to 980 m/z. The isolated ions were fragmented using HCD with 25% Normalized Collision Energy. Other settings for DIA include an RF lens of 40%, normalize ACG target of 500% (5E4), and a maximum injection time of 3 ms.

#### LC-MS/MS proteomics data acquisition for mammalian mitochondrial samples

Peptides were resuspended in 0.2% formic acid, and the concentration of each sample was determined using the Pierce Quantitative Fluorescent Peptide Assay (Thermo Scientific). LC separation was performed using the Thermo Vanquish Neo UHPLC system. A 25 cm Aurora Ultimate XT C18 UHPLC column (250 mm × 150 µm, 1.7 µm, IonOpticks) was used with a 36 min gradient using mobile phase A consisting of 0.1% formic acid in H_2_O, and mobile phase B consisting of 0.1% formic acid in ACN:H_2_O (80:20, v/v). An EASY-Spray source was used and the temperature was set at 40 °C. Each sample was first held at 3% B for 1 min with an 800 nL/min flow rate, then the gradient was increased to 8% B over 0.1 min, then 11.5% B over 2.8 min. The flow rate was dropped to 600 nL/min and increased to 11.6% B over 0.1 min, before increasing to 43.8% B over 26.6 min. The flow rate was adjusted to 800 nL/min and increased to 99% B over 1 min, followed by 4.3 min at 99% for column washing. An Acclaim PepMap C18 HPLC trap column (20 mm × 75 µm, 3 µm) was used for sample loading. MS detection was performed with a Thermo Astral mass spectrometer in positive mode. The source voltage was 1.5 kV, ion transfer tube temperature was set to 280 °C, and RF lens was at 40%. Full MS spectra were acquired from m/z 380 to 980 at the Orbitrap resolution of 240,000, with the normalized AGC target of 500% (5E6) and a maximum injection time of 3 ms. Data-independent acquisition (DIA) was performed with the Astral analyzer with 300 isolation windows of 2-Th scanning from 380 to 980 m/z. The isolated ions were fragmented using HCD with 25% Normalized Collision Energy. Other settings for DIA include an RF lens of 40%, normalize ACG target of 500% (5E4), and a maximum injection time of 3 ms.

#### Data analysis

Raw files were analyzed by the CHIMERYS search algorithm with INFERYS Rescoring incorporated in Proteome Discoverer v.3.2.0.450 software against yeast or human databases downloaded from Uniprot. MS2 Apex (quan in all files) was used as the quantification method for the searches.

### LC-MS/MS lipidomics

#### Yeast growth for de novo CoQ measurements

Starter cultures (SC 2% glucose, w/v, 3 mL) were inoculated with individual colonies and incubated overnight (30 °C, 230 rpm, 14–16 h). Next, 50 mL cultures in SC pABA^–^ (2% glucose, w/v) were inoculated with 2.5 × 10^7^ cells and incubated (30 °C, 230 rpm) until reaching an OD_600_ ~ 2. The cells were then centrifuged (3000 × *g*, 3 min, r.t.) and resuspended in SC pABA^–^ media (3% glycerol, w/v) containing ^13^C_6_−4HB (50 μM, Sigma) and incubated (30 °C, 230 rpm). At 2, 4 and 6 h after the media swap, 1 × 10^8^ cells were collected, snap-frozen in LN_2_, and stored at −80 °C.

#### Lipid extraction

Lipids from cell pellets or pure mitochondrial samples were extracted using MTBE (Sigma-Aldrich). Frozen cell pellets were resuspended in 225 μL 100% LC-grade methanol (Fisher), containing 1 μM CoQ_8_ (Avanti Lipids) as an internal standard. Glass beads (100 μL, 0.5 mm; BioSpec) were then added and the samples were vortexed using a Vortex Genie (10 min, 3000 rpm, 4 °C) to lyse the cells. Next, 187.5 μL of water and 750 μL of MTBE were added to each sample and the tubes were vortexed again for (3 min, 3000 rpm, 4 °C). To separate the layers, samples were centrifuged (3 min, 1000 × *g*, 4 °C). The organic (top) layer was removed into a separate microcentrifuge tube and a new 750 μL of MTBE was added. Organic extraction was repeated a second time with the second MTBE layer added to the first. Samples were dried by vacuum centrifugation and resuspended in 50 μL of 20 mM ammonium acetate in 78% (v/v) methanol, 20% IPA (Sigma-Aldrich) and 2% water.

#### Untargeted LC-MS/MS lipidomics data acquisition

A Vanquish Horizon UHPLC system (Thermo Scientific) connected to an Exploris 240 Orbitrap mass spectrometer (Thermo Scientific) was used for LC-MS analysis. A Waters Acquity CSH C18 column (100 mm × 2.1 mm, 1.7 μm) was held at 50 °C with the flow rate of 0.4 mL/min for lipid separation. A Vanquish binary pump system was employed to deliver mobile phase A, consisting of 5 mM ammonium acetate in ACN/H_2_O (70/30, vol/vol) containing 250 μL/L acetic acid, and mobile phase B consisting of 5 mM ammonium acetate in IPA/ACN (90/10, vol/vol) containing 250 μL/L acetic acid. The gradient was set as follows: B was at 2% for 2 min and increased to 30% over the next 1 min, then further ramped up to 50% within 1 min and to 85% over the next 14 min, and then raised to 99% over 1 min and held for 7 min, before re-equilibration for 4 min at 2% B. Samples were injected by a Vanquish Split Sampler HT autosampler (Thermo Fisher Scientific), while the autosampler temperature was kept at 4 °C. Samples were ionized by a heated ESI source with a vaporizer temperature of 200 °C. The sheath gas was set to 25 units, auxiliary gas to 15 units and the sweep gas to 2 unit. For untargeted discovery lipidomics, the MS was operated in polarity switching mode with the spray voltage set to 3500 V for positive mode and 2500 V for negative mode. The inlet ion transfer tube temperature was kept at 300 °C with 70% RF lens. Full MS1 scans were acquired at 22,500 resolution (at 200 m/z), a max ion accumulation time of 100 ms and with a scan range of m/z 200–1,600. MS2 scans (top 3) were acquired at 30,000 resolution (at 200 m/z), max ion accumulation time of 50 ms, a 1.0 m/z isolation window, stepped NCE at 20%, 30% and 40%, and a 10.0 s dynamic exclusion. Automatic gain control (AGC) targets were set to standard mode for both MS1 and MS2 acquisitions.

#### Targeted LC-MS/MS lipidomics data acquisition

A Vanquish Horizon UHPLC system (Thermo Scientific) connected to an Exploris 240 Orbitrap mass spectrometer (Thermo Scientific) was used for LC-MS analysis. A Waters Acquity CSH C18 column (100 mm × 2.1 mm, 1.7  μm) was held at 35 °C with the flow rate of 0.3 mL/min for lipid separation. A Vanquish binary pump system was employed to deliver mobile phase A, consisting of 5 mM ammonium acetate in ACN/H_2_O (70/30, vol/vol) containing 250 μL/L acetic acid, and mobile phase B consisting of 5 mM ammonium acetate in IPA/ACN (90/10, vol/vol) containing 250 μL/L acetic acid. The gradient was set as follows: B was at 2% for 2 min and increased to 30% over the next 3 min, then further ramped up to 50% within 1 min and to 85% over the next 14 min, and then raised to 99% over 1 min and held for 4 min, before re-equilibration for 5 min at 2% B. Five microliters of the sample were injected by a Vanquish Split Sampler HT autosampler (Thermo Fisher Scientific), while the autosampler temperature was kept at 4 °C. Samples were ionized by a heated ESI source with a vaporizer temperature of 200 °C. The sheath gas was set to 40 units, auxiliary gas to 8 units and the sweep gas to 1 unit. The ion transfer tube temperature was kept at 300 °C with a 70% RF lens. The spray voltage was set to 3,500 V for positive mode and 2,500 V for negative mode. For CoQ intermediate analysis, targeted acquisition was performed using parallel reaction monitoring mode with polarity switching, targeting scans to CoQ_6_ (m/z 591.4408), ^13^C_6_-CoQ_6_ (m/z 597.4609), DMQ_6_ (m/z 561.4302), CoQ_10_ (m/z 863.6912) and CoQ_8_ (m/z 727.5660) in positive polarity, and PPHB_6_ (m/z 545.4000) and ^13^C_6_-PPHB_6_ (m/z 551.4201) in negative polarity. For NAPE analysis, targeted acquisition was performed using parallel reaction monitoring mode with polarity switching, targeting scans to NAPE 50:1 (m/z 954.7532), NAPE 52:1 (m/z 982.7845), NAPE 52:2 (m/z 980.7689), NAPE 52:3 (m/z 978.7532), NAPE 54:1 (m/z 1010.8158), NAPE 54:2 (m/z 1008.8002), NAPE 54:3 (m/z 1006.7845), NAPE 56:1 (m/z 1038.8471), NAPE 56:2 (m/z 1036.8315), NAPE 56:5 (m/z 1030.7845), and NAPE 56:6 (m/z 1028.7689) in negative polarity. MS acquisition parameters include resolution of 15,000, an isolation window of 2 m/z, stepped HCD energies of 20%, 40% and 60% for positive mode or 25%, 30%, and 40% for negative mode, standard AGC target and auto maximum ion injection time.

#### Data analysis

For untargeted lipidomic analyses, LC–MS files were processed using Compound Discoverer 3.2 (Thermo Scientific) and either LipiDex1.0 or Lipidex2.0^60^. All peaks with a 1.4–23 min retention time and 100–5,000 Da MS1 precursor mass were aggregated into compound groups using a 10 ppm mass tolerance and 0.4 min retention time tolerance. Peaks were excluded if peak intensity was less than 2 × 10^6^, peak width was greater than 0.75 min, signal-to-noise ratio was less than 1.5 or intensity was <3-fold greater than the blank. MS2 spectra were searched against an in silico generated spectral library^61^. Spectra with a dot product score >500 and a reverse dot product score >700 were retained for further analysis. Lipid MS/MS spectra that contained <75% interference from co-eluting isobaric lipids, eluted within a 3.5 median absolute retention time deviation of each other and were found within at least four processed files were used for identification at the individual fatty acid substituent levels of structural resolution. If individual fatty acid substituents were unresolved, then identifications were made with the sum of the fatty acid substituents. Lipid identifications were filtered with the Degreaser module within LipidDex2 (v0.1.0)^62^, based on retention time modeling. The retention time tolerance used was 0.5 min. Unreliable identifications were discarded.

Targeted quantitative analysis of all acquired compounds was processed using TraceFinder 5.1 (Thermo Scientific) with a mass accuracy of 5 ppm. The result of peak integration was manually examined.

### Immunoblotting

#### Antibodies

Primary antibodies used in this study include anti-Kar2 (SCBT sc-33630, 1:5000), and anti-Cit1^27^ (custom made at Biomatik, 1:4000), anti-Por1 (Abcam ab110326, 1:1000), anti-Pma1 (Abcam ab4645, 1:1000), and anti-NeonGreen (Proteintech 29523-1-AP, 1:1000). Secondary antibodies include anti-mouse immunoglobulin-G (IgG) horseradish peroxidase (HRP)-linked (Cell Signaling Technology #7076, 1:5000) and anti-rabbit IgG HRP-linked (Cell Signaling Technology #7074, 1:5,000).

#### Mitochondrial isolation, western blotting

Spheroplast, crude mitochondria, and pure mitochondria samples were collected throughout the mitochondrial isolation process. Samples were solubilized in RIPA buffer and protein concentrations were determined by BCA protein assay (Thermo). Protein (10 μg) was loaded onto NuPAGE 4–12% Bis-Tris gels (Thermo) and separated (200 V, 35 min). Proteins were transferred to a polyvinylidene fluoride (PVDF) membrane (Sigma) and blocked with 5% non-fat dry milk (NFDM) in tris-buffered saline with 0.1% Tween-20 (TBST) (1 h, r.t.). The membranes were then probed with primary antibodies diluted in 5% NFDM in TBST (overnight, 4 °C). Membranes were washed three times with TBST and then probed with secondary antibodies diluted in 5% NFDM in TBST (1 h, r.t.). Membranes were washed three times with TBST and developed by enhanced chemiluminescence (ECL) using the SuperSignal West Dura substrate (Thermo, 34075). Developed membranes were imaged on a ChemiDoc system running Image Lab Touch Software 3.0.1.14 (Bio-Rad).

### Protein purification

#### NAPE-PLD

NAPE-PLD was purified as described previously^63^ with some modifications. *E. coli* BL21-CodonPlus (DE3)-RIPL cells (Thermo) harboring plasmid with His_8_-MBP-NAPE-PLD NΔ46 were grown in a 2 L LB culture at 37 °C to an OD_600_ of 0.6 and expression was induced with 1 mM isopropyl-B-D-thiogalactoside (IPTG) at 22 °C for 16 h. The cells were harvested by centrifugation (5000 × *g*, 10 min, 4 °C) and stored at −80 °C. The cell pellet was resuspended in 35 mL Lysis Buffer (20 mM HEPES pH 7.4, 200 mM NaCl, 0.1% Triton X-100, 1 Roche protease COmplete inhibitor tablet, 1 mg/mL lysozyme) and lysed via sonication on ice (5 rounds of 20 s on, 60 s off, 75% amplitude). The lysate was clarified by centrifugation (15,000 × *g*, 30 min, 4 °C). The cleared lysate was mixed with amylose resin (New England Biolabs) and incubated on a rotor (2 h, 4 °C). The resin was washed five times with Wash Buffer (20 mM HEPES pH 7.4, 200 mM NaCl, 1 mM EDTA, 0.05% Triton X-100) and the protein was eluted with Wash Buffer containing 10 mM maltose. The protein concentration was determined by BCA protein assay (Thermo) and protein was snap frozen and stored at −80 °C.

#### Fmp30

*E. coli* OverExpress C41(DE3) cells (Sigma) harboring plasmid with His_8_-MBP-Fmp30 NΔ76 (wild-type or H265N) were grown in a 2 L LB culture at 37 °C to an OD_600_ of 0.6 and expression was induced with 1 mM IPTG at 22 °C for 16 h. The cells were harvested by centrifugation (5000 × *g*, 10 min, 4 °C) and stored at −80 °C. The cell pellet was resuspended in 35 mL Lysis Buffer (50 mM HEPES pH 7.4, 250 mM NaCl, 5% glycerol, 0.5% DDM, 1 Roche protease COmplete inhibitor tablet, 1 mg/mL lysozyme) and lysed via sonication on ice (5 rounds of 20 s on, 60 s off, 75% amplitude). The lysate was clarified by centrifugation (15,000xg, 30 min, 4 °C). The cleared lysate was mixed with amylose resin (New England Biolabs) and incubated on a rotor (2 h, 4 °C). The resin was washed five times with Wash Buffer (50 mM HEPES pH 7.4, 250 mM NaCl, 5% glycerol, 0.05% DDM) and the protein was eluted with Wash Buffer containing 10 mM maltose. The eluted protein was concentrated with a MW-cutoff spin filter (50 kDa MWCO) and subjected to size exclusion chromatography on a HighLoad 16/600 Superdex 200 pg (GE Healthcare) and eluted with SEC buffer (0.025% DDM, 50 mM HEPES pH 7.4, 250 mM NaCl, 5% glycerol). Fractions containing MBP-Fmp30, assessed by SDS-PAGE, were pooled and concentrated with a MW-cutoff spin filter. The protein concentration was determined by BCA protein assay (Thermo) and protein was snap frozen and stored at −80 °C.

### Enzyme activity assays

#### Bis-pNPP phosphodiesterase assay

Phosphodiesterase activity was measured via hydrolysis of the phosphodiester bond of the generic colorimetric reagent Bis(p-nitrophenyl phosphate (Bis-pNPP, Sigma). Protein (10 μg) was incubated with Bis-pNPP (25 mM) in 100 μL assay buffer (50 mM Tris pH 8.5, 0.1% DDM, 1 mM MnCl_2_, 1 mM CoCl_2_). The reaction was monitored by increase in absorbance in Epoch2 plate reader (BioTek), with OD_410_ measured every minute.

#### Enzyme assay and lipid extraction

All lipid substrates (18:1 PE-N-18:1, 18:1 PA, 18:1 (Δ9-Cis) PE, 18:1 (Δ9-Cis PC), 18:1 CL, 18:1 PI) were purchased from Avanti. The activity of recombinant MBP-Fmp30 NΔ76 or MBP-NAPE-PLD NΔ46 was measured by incubating the protein (10 μg) with 1 μM of the substrate in 187.5 μL assay buffer (50 mM Tris pH 8.5, 0.1% DDM, 1 mM MnCl_2_, 1 mM CoCl_2_) for 1 h at 37 °C. For reactions using mitochondria purified from *fmp30*Δ as a substrate, mitochondria (25 μg) were first solubilized by incubation with 1 g/g DDM (detergent/protein w/w) in solubilization buffer (50 mM NaCl, 50 mM imidazole, 2 mM 6-aminohexanoic acid, 1 mM EDTA, pH 7.0). Solubilized mitochondria were diluted in assay buffer with 20 μg protein and incubated for 1 h at 37 °C. The reactions were terminated by adding 225 μL of methanol containing 100 pmol [^2^H_4_]-palmitoylethanolamide (Caymen Chemical) as the internal standard. Lipid extraction was conducted with MTBE as described above.

#### Targeted LC-MS/MS lipidomics data acquisition

A Vanquish Horizon UHPLC system (Thermo Scientific) connected to an Exploris 240 Orbitrap mass spectrometer (Thermo Scientific) was used for LC-MS analysis. A Waters Acquity CSH C18 column (100 mm × 2.1 mm, 1.7 μm) was held at 55 °C with the flow rate of 0.3 mL/min for lipid separation. A Vanquish binary pump system was employed to deliver mobile phase A, consisting of 2.5 mM ammonium bicarbonate in IPA/ACN/H_2_O (15/35/50, vol/vol), and mobile phase B consisting of 2.5 mM ammonium bicarbonate in IPA/ACN/H_2_O (70/25/5, vol/vol). The gradient was set as follows: B started at 20% and increased to 45% over the next 2.5 min, then further ramped up to 65% within 7 min and to 85% over the next 3 min, and then raised to 99% over 6 min and held for 3 min, before re-equilibration for 4 min at 20% B. Five microliters of the sample were injected by a Vanquish Split Sampler HT autosampler (Thermo Fisher Scientific), while the autosampler temperature was kept at 4 °C. Samples were ionized by a heated ESI source with a vaporizer temperature of 200 °C. The sheath gas was set to 25 units, auxiliary gas to 15 units and the sweep gas to 5 unit. The ion transfer tube temperature was kept at 300 °C with a 70% RF lens. The spray voltage was set to 2600 V for positive mode and 2100 V for negative mode. Targeted acquisition was performed using parallel reaction monitoring mode with polarity switching, targeting scans to 32:0 PA (m/z 647.4657), 32:1 PA (m/z 645.4501), 32:2 PA (m/z 643.4344), 34:1 PA (m/z 673.4814), 34:2 PA (m/z 671.4657), 36:1 PA (m/z 701.5127), 18:1 PI (m/z 861.5499), 18:1 (Δ9-Cis) PE (m/z 742.5392), and 18:1 (Δ9-Cis) PC (m/z 784.5862) in negative polarity and [^2^H_4_]-palmitoylethanolamide (m/z 304.3148), 18:1 CL (m/z 1475.0687), 18:1 PE-N-18:1 (m/z 1008.7991) and 18:1 NAE (m/z 326.3054) in positive polarity. MS acquisition parameters include resolution of 30,000, an isolation window of 2 m/z, stepped HCD energies of 25%, 30%, and 40%, standard AGC target and auto maximum ion injection time.

#### Data analysis

Targeted quantitative analysis of all acquired compounds was processed using TraceFinder 5.1 (Thermo Scientific) with a mass accuracy of 5 ppm. The result of peak integration was manually examined.

### Statistics and reproducibility

All experiments were performed in at least biological triplicate, unless otherwise stated. No statistical methods were used to predetermine sample sizes but our sample sizes are similar to those reported in previous publications^64,65^. Data distribution was assumed to be normal, but this was not formally tested. No randomization was used for experimental groups. Data collection and analysis were not performed blind to the conditions of the experiments. No data were excluded from the analyses. For all assays, quantification and statistics were derived from *n* = 3 independent biological replicates unless specified in the legends. All statistical analysis was performed using Excel, GraphPad Prism, or Python. All results are presented as the arithmetic mean ± s.d. *P* values were calculated using either unpaired, two-sided Student’s *t* test, two-sided Welch’s *t*-test, or Brown-Forsythe and Welch one-way ANOVA tests as specified in the methods and legends. *P* values less than 0.05 were considered significant.

### Reporting summary

Further information on research design is available in the Nature Portfolio Reporting Summary linked to this article.

## Supplementary information

Supplementary Information

Reporting Summary

Transparent Peer Review file

## Source data

Source Data

## Data Availability

All mass spectrometry data (proteomics and lipidomics) have been deposited in MassIVE with the primary accession code MSV000100896. All other data generated in this study are provided in the Source Data file. Source data are provided with this paper.
