# Supplementary material for: Fmp30p is a mitochondrial phosphatidylinositol hydrolase that modulates CoQ biosynthesis
Source: Nat Commun. 2026 May 30;17:7013. doi: 10.1038/s41467-026-73766-x (PMC13392021; doi:10.1038/s41467-026-73766-x)
Supplement: Supplementary file 1 — Supplementary Information [file 41467_2026_73766_MOESM1_ESM.pdf]

SUPPLEMENTARY INFORMATION

Supplementary Figures

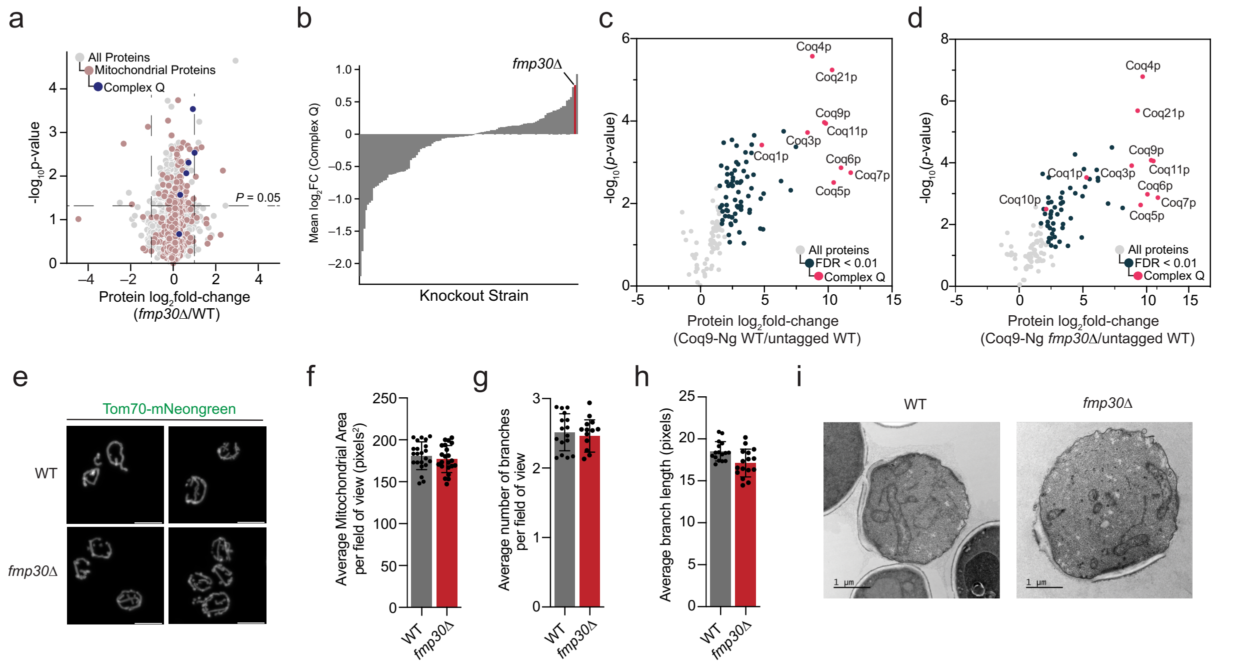

Supplementary Fig. 1. a, Relative protein abundances of *fmp30Δ* compared to WT versus statistical significance. Mitochondrial proteins are highlighted in light red, complex Q proteins are highlighted in dark blue. **b**, Rank ordering of gene deletion strains in the Y3K dataset[1] based on their mean complex Q protein abundance. (**c-d**) Relative protein abundance versus statistical significance of proteins enriched following mitochondrial crosslinking and immunoprecipitation of endogenously expressed Coq9-mNeonGreen in a WT strain (**c**) or *fmp30Δ* strain (**d**) compared to a WT strain expressing no tagged bait. **e**, Relative max projection z-stack images of WT and *fmp30Δ* expressing endogenously-tagged Tom70-mNeonGreen. Scale bar = 5 μm. (**f-h**) Quantification of the average mitochondrial area (**f**), average number of mitochondrial branches per field of view (**g**), or average branch length (**h**) of the images in **e**. **i**, Representative transmission electron microscopy of WT and *fmp30Δ* yeast. Scale bar = 1 μm. For **a**, **c-d**, n = 3 biological replicates, two-sided Student's *t*-test. For **f-h**, data are displayed as mean ± s.d., n>100 cells from three independent experiments, Welch's *t*-test. Source data are provided as a Source Data file.

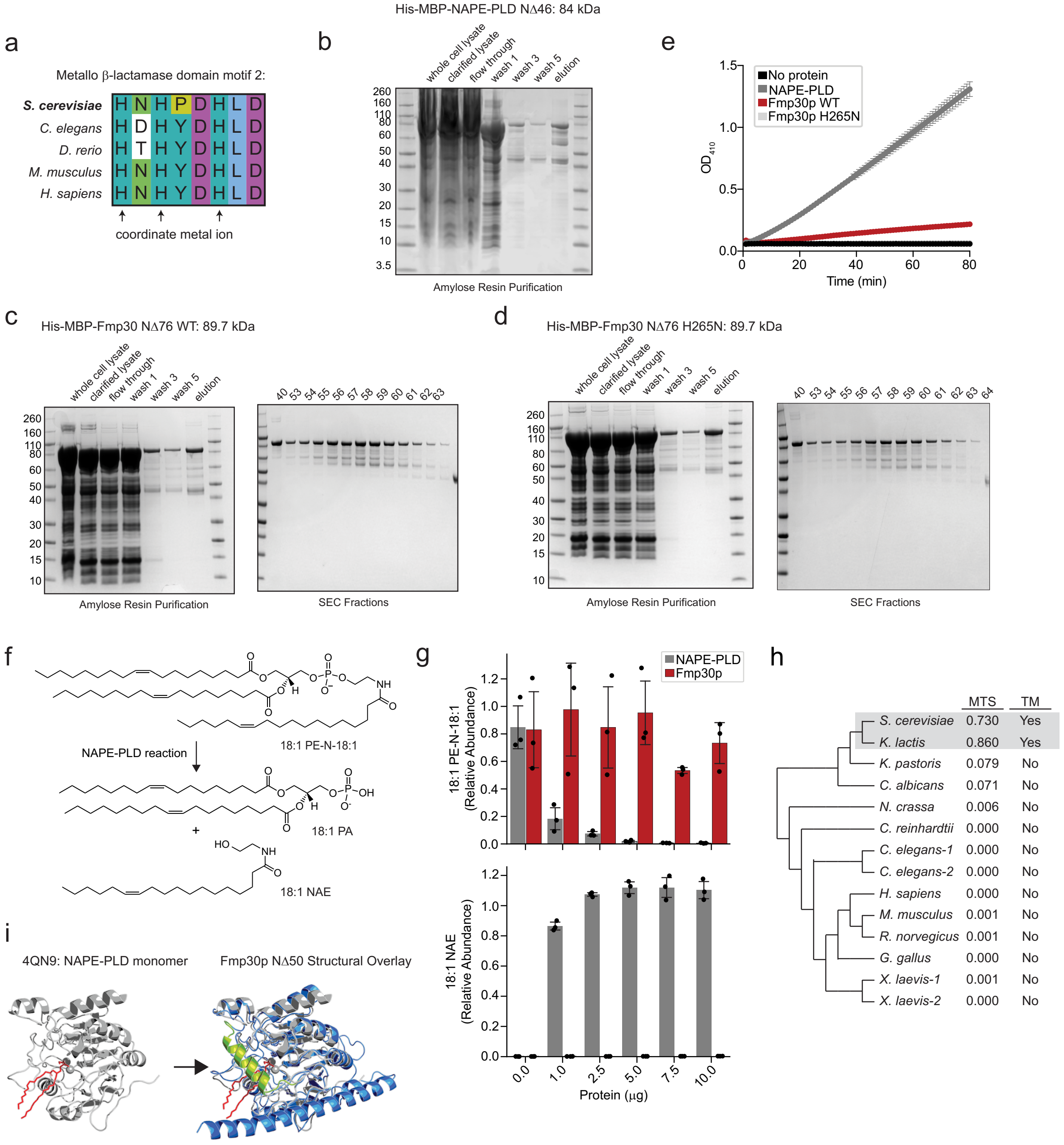

Supplementary Fig. 2. Biochemical validation of NAPE-PLD and Fmp30p phosphodiesterase activity. **a**, Multiple sequence alignment for metallo-β-lactamase motif 2, highlighting the metal binding site, in NAPE-PLD homologs from various species. Colors represent default ClustalX amino acid class color scheme. (**b-d**) SDS-PAGE gels of purification of recombinant MBP-NAPE-PLD NΔ46 WT (**b**), MBP-Fmp30 NΔ76 WT (**c**), and MBP-Fmp30 NΔ76 H265N (**d**). **e**, Phosphodiesterase activity of NAPE-PLD, Fmp30p WT, and Fmp30p H265N as measured by colorimetric reaction with the generic substrate Bis-pNPP. **f**, Phospholipase D enzymatic reaction catalyzed by NAPE-

PLD. PE-N, n-acyl phosphatidylethanolamine; PA, phosphatidic acid; NAE, N-acylethanolamide. **g,** Relative abundance of 18:1 PE-N-18:1 (18:1 NAPE, top) or 18:1 NAE (bottom) generated after incubation of 18:1 PE-N-18:1 with the indicated concentration of purified recombinant Fmp30p or NAPE-PLD for 30 min. NAE, N-acylethanolamide. Related to data shown in **Fig 2b**-**c. h,** Phylogenetic analysis of *FMP30* homologs. Sequences were analyzed using MitoFates to determine mitochondrial targeting sequence (MTS) probability and AlphaFold structures were analyzed for presence of a transmembrane (TM) domain. **i,** Crystal structural of NAPE-PLD (gray, PDB:4QN9) with PE (red, left) and overlaid with the AlphaFold structure of Fmp30p (right). Variable alpha helix in Fmp30p is colored green. For **g**, data are displayed as mean ± s.d., n = 3 biological replicates. Source data are provided as a Source Data file.

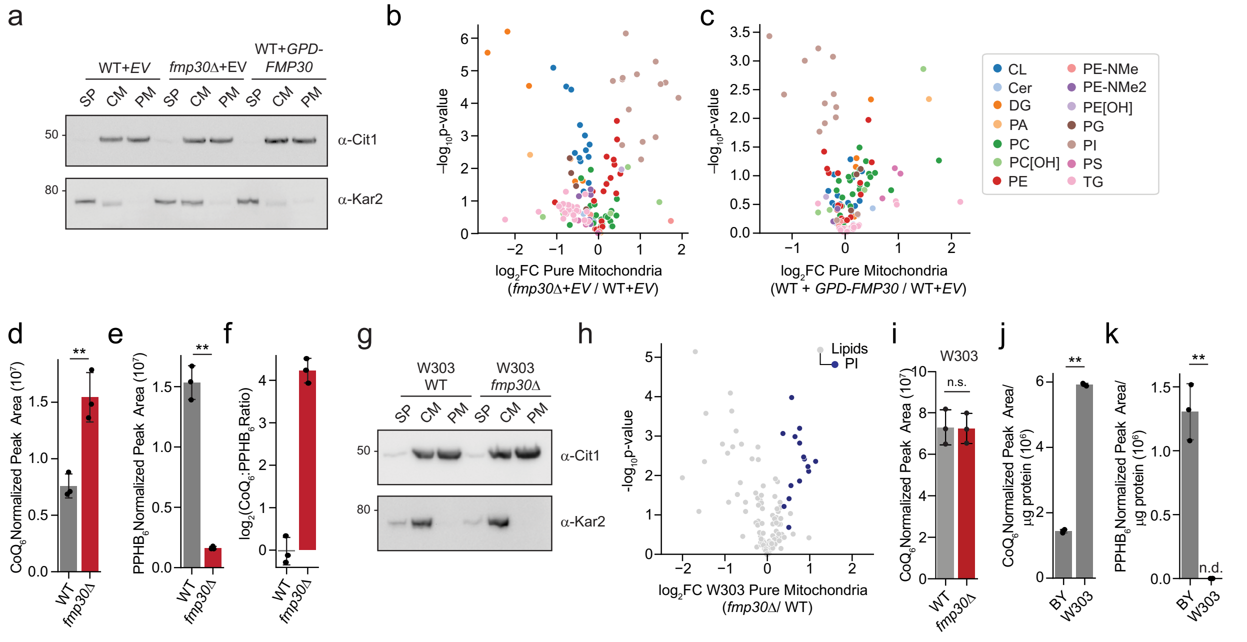

Supplementary Fig. 3. a, Western blot of subcellular samples derived from fractionated WT + *EV*, *fmp30Δ* + *EV*, and WT + *GPD-FMP30* yeast. SP, spheroplast; CM, crude mitochondria; PM, pure mitochondria. Kar2, endoplasmic reticulum; Cit1, mitochondria. A representative Western blot from 4 independent experiments. (**b-c**) Data from **Fig 2 h-i** showing relative lipid abundance in purified mitochondria from *fmp30Δ* + *EV* (**b**) or WT + *GPD-FMP30* (**c**) compared to WT + *EV*, versus statistical significance. Points are color-coded according to lipid class. (**d-f**) Abundance of CoQ_6 (**d**) $PPHB_6$ (**e**), and the Log_2 transformed ratio of CoQ_6 to $PPHB_6$ abundance (normalized to WT) (**f**) in BY4742 WT and *fmp30Δ* yeast grown in fermentative media. **g**, Western blot of subcellular samples derived from W303 WT and *fmp30Δ*. SP, spheroplast; CM, crude mitochondria; PM, pure mitochondrial. Kar2, endoplasmic reticulum; Cit1, mitochondria. **h,** Relative lipid abundance in purified mitochondrial from W303 *fmp30Δ* compared to W303 WT, versus statistical significant. PI species are colored in dark blue. **i,** Abundance of CoQ_6 in pure mitochondria isolated from W303 WT and *fmp30Δ*. (**j-k**) Abundance of CoQ_6 (**j**) and $PPHB_6$ (**k**) from WT BY4742 and W303 strains. For **b-c**, n = 4 independent biological experiments. For **d-f** and **h-k**, data are displayed as mean ± s.d., n = 3 biological replicates, two-sided Student's *t*-test (**d**, $^{**}P=5.02\times10^{-3}$; **e**, $^{**}P=7.18\times10^{-5}$; **j**, $^{**}P=2.82\times10^{-8}$; **k**, $^{**}P=5.01\times10^{-4}$). Source data are provided as a Source Data file.

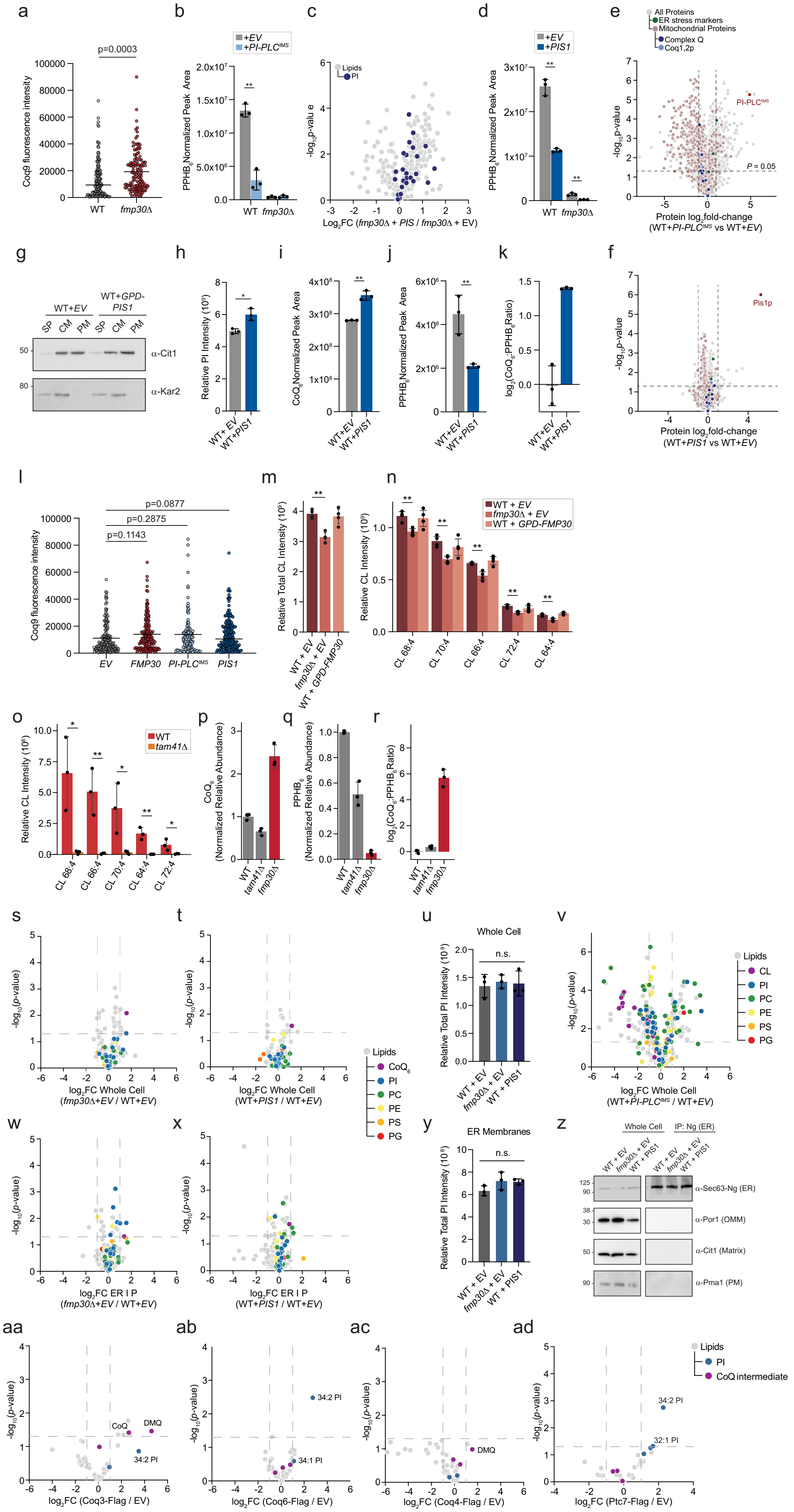

a
p=0.0003
Coq9 fluorescence intensity
WT
fmp30Δ
b
+EV
+PI-PLC^IMS
PPHB6 Normalized Peak Area
c
Lipids
PI
-log10 p-value
Log2FC (fmp30Δ + PIS / fmp30Δ + EV)
d
+EV
+PIS1
PPHB6 Normalized Peak Area
e
All Proteins
ER stress markers
Mitochondrial Proteins
Complex Q
Coq1,2p
PI-PLC^IMS
P = 0.05
-log10 p-value
Protein log2fold-change
(WT+PI-PLC^IMS vs WT+EV)
g
WT+EV
WT+GPD-PIS1
SP CM PM SP CM PM
α-Cit1
α-Kar2
h
Relative PI Intensity (10^9)
WT+ EV
WT+PIS1
i
CoQ6 Normalized Peak Area
j
PPHB6 Normalized Peak Area
k
log2(CoQ6:PPHB6 Ratio)
f
Pis1p
Protein log2fold-change
(WT+PIS1 vs WT+EV)
l
p=0.0877
p=0.2875
p=0.1143
Coq9 fluorescence intensity
EV
FMP30
PI-PLC^IMS
PIS1
m
Relative Total CL Intensity (10^9)
WT + EV
fmp30Δ + EV
WT + GPD-FMP30
n
WT + EV
fmp30Δ + EV
WT + GPD-FMP30
Relative CL Intensity (10^9)
CL 68:4
CL 70:4
CL 66:4
CL 72:4
CL 64:4
o
WT
tam41Δ
Relative CL Intensity (10^8)
CL 68:4
CL 66:4
CL 70:4
CL 64:4
CL 72:4
p
CoQ6
(Normalized Relative Abundance)
WT
tam41Δ
fmp30Δ
q
PPHB6
(Normalized Relative Abundance)
r
log2(CoQ6:PPHB6 Ratio)
s
-log10(p-value)
log2FC Whole Cell
(fmp30Δ+EV / WT+EV)
t
log2FC Whole Cell
(WT+PIS1 / WT+EV)
Lipids
CoQ6
PI
PC
PE
PS
PG
u
Whole Cell
n.s.
Relative Total PI Intensity (10^9)
WT + EV
fmp30Δ + EV
WT + PIS1
v
Lipids
CL
PI
PC
PE
PS
PG
log2FC Whole Cell
(WT+PI-PLC^IMS / WT+EV)
w
log2FC ER I P
(fmp30Δ+EV / WT+EV)
x
log2FC ER I P
(WT+PIS1 / WT+EV)
y
ER Membranes
n.s.
Relative Total PI Intensity (10^9)
z
Whole Cell
IP: Ng (ER)
α-Sec63-Ng (ER)
α-Por1 (OMM)
α-Cit1 (Matrix)
α-Pma1 (PM)
aa
CoQ
DMQ
34:2 PI
log2FC (Coq3-Flag / EV)
ab
34:2 PI
34:1 PI
log2FC (Coq6-Flag / EV)
ac
DMQ
log2FC (Coq4-Flag / EV)
ad
Lipids
PI
CoQ intermediate
34:2 PI
32:1 PI
log2FC (Ptc7-Flag / EV)

Supplementary Fig. 4. **a**, Quantification of total Coq9p fluorescence intensity per cell of the cells imaged in **Fig 3a**. **b,** Abundance of $PPHB_6$ from WT expressing *PI-PLC^{IMS}*, related to **Fig 3e**-**f**. **c**, Relative lipid abundance from *fmp30*Δ expressing *PIS1* compared to *fmp30*Δ carrying an *EV*, versus statistical significance. **d**, Abundance of $PPHB_6$ from WT expressing *PIS1*, related to **Fig 3g**-**h**. (**e**-**f**) Relative protein abundances of WT yeast expressing *PI-PLC^{IMS}* (**e**) or *PIS1* (**f**) compared to WT harboring an empty vector, versus statistical significance. Mitochondrial proteins are highlighted in light red, complex Q proteins are highlighted in dark blue, and ER stress markers are highlighted in dark green. **g**, Western blot of subcellular samples derived from fractionated WT + *EV* and WT + *PIS1* yeast. SP, spheroplast; CM, crude mitochondria; PM, pure mitochondria. Kar2, endoplasmic reticulum; Cit1, mitochondria. A representative Western blot from 3 independent experiments. (**h**-**k**) Abundance of total PI (**h**), CoQ_6 (**i**), $PPHB_6$ (**j**), and the Log_2 transformed ratio of CoQ_6 to $PPHB_6$ abundance (normalized to WT) (**k**) of purified mitochondria from WT expressing an empty vector or *PIS1*. **l**, Quantification of total Coq9p fluorescence intensity per cell of the cells imaged in **Fig 3i. (m**-**n**) Normalized abundance of total cardiolipin (CL) (**m**) and individual remodeled CL species (**n**) in pure mitochondria isolated from WT + *EV*, *fmp30*Δ + *EV*, and WT + *FMP30* yeast from **Fig 2 h-i**. **o**, Normalized abundance of individual remodeled CL species from WT and *tam41*Δ cells. (**p**-**r**) Relative CoQ_6 abundance (**p**), $PPHB_6$ abundance (**q**), and the Log_2 transformed ratio of CoQ_6 to $PPHB_6$ abundance (**r**) in *tam41*Δ and *fmp30*Δ normalized to WT. (**s**-**t**) Relative whole cell lipid abundance from *fmp30*Δ + *EV* (**s**) and WT + *PIS1* (**t**) compared to WT + *EV*, versus statistical significance. Points are color-coded according to lipid class. **u**, Relative total abundance of whole cell PI in WT + *EV*, *fmp30*Δ + *EV*, and WT + *PIS1* yeast strains. **v**, Relative lipid abundance from WT expressing *PI-PLC^{IMS}* compared to WT carrying an *EV*, versus statistical significance. Points are color-coded according to lipid class. (**w**-**x**) Relative ER lipid abundance obtained using immunoprecipitation from *fmp30*Δ + *EV* (**w**) and WT + *PIS1* (**x**) compared to WT + *EV*, versus statistical significant. Points are color-coded according to lipid class. **y**, Relative total abundance of ER PI in WT + *EV*, *fmp30*Δ + *EV*, and WT + *PIS1* yeast strains. **z**, Western blot of ER membranes following Sec63-NeonGreen immunoprecipitation. Sec63, endoplasmic reticulum; Por1, outer mitochondrial membrane; Cit1, mitochondrial matrix; Pma1, plasma membrane. (**aa**-**ad**) Relative lipid abundance versus statistical significance of lipids enriched following mitochondrial isolation and FLAG immunoprecipitation in a WT strain overexpressing *COQ3*-FLAG (**aa**), *COQ6*-FLAG (**ab**), *COQ4*-FLAG (**ac**) or *PTC7*-FLAG (**ad**) compared to WT expressing an empty vector. PI species are highlighted in blue, CoQ intermediates are highlighted in purple. For **a**, all data points are displayed with the median indicated, n>100 cells from three independent experiments, Welch's *t*-test. For **l**, all data points are displayed with the median indicated, n>100 cells from three independent experiments, Brown-Forsythe and Welch ANOVA tests. For **b**-**f**, **h**-**k**, **m**-**y**, and **aa**-**ad**, data are

displayed as mean ± s.d., n=3 biologically independent samples, two-sided Student's *t*-test (**b**, **P=5.31x10^{-4}; **d**, **P=1.97x10^{-4}, *P=1.94x10^{-3}; **h**, **P=1.07x10^{-2}; **i**, **P=7.93x10^{-4}; **j**, **P=9.97x10^{-3}; **m**, **P=3.93x10^{-4}). Otherwise, P value is indicated or for more than four comparisons *$P<0.05$, **$P<0.01$. Source data are provided as a Source Data file.

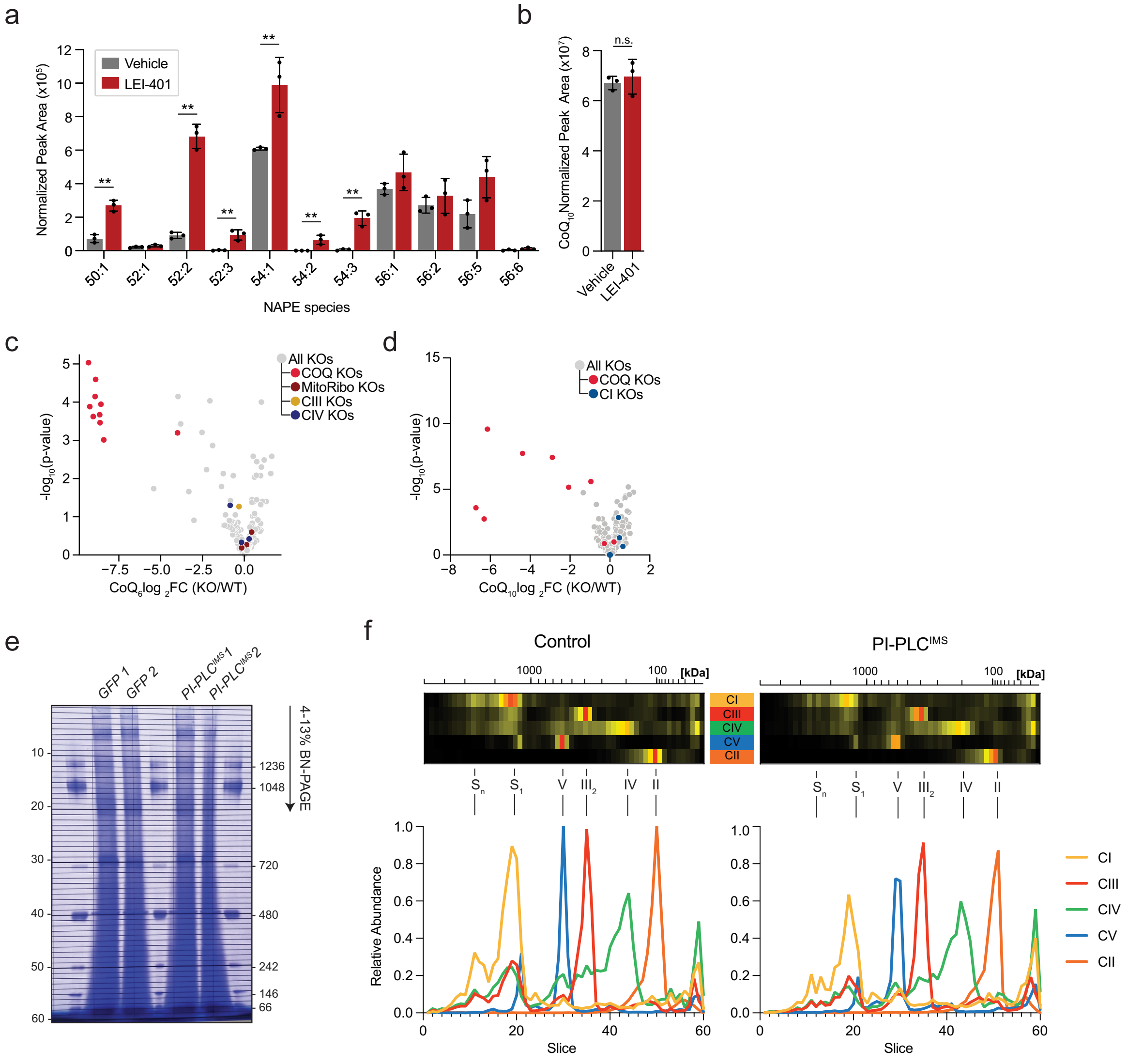

Supplementary Fig. 5. (**a-b**) Relative abundances of the indicated NAPE species (**a**) or CoQ_{10} (**b**) of U2OS cells treated with the NAPE-PLD inhibitor LEI-410 (20 µM, 24 h) or vehicle. **c**, CoQ_6 abundance across all yeast gene deletions analyzed in the Y3K study[1], versus statistical significance. **d**, CoQ_{10} abundance across all HAP1 deletion cell lines analyzed in the MITOMICs study[2], versus statical significance. **e**, Blue native PAGE of digitonin-solubilized mitochondria isolated from U2OS cells stably expressing *GFP* or *PI-PLC*^{IMS}. Gel lanes were cut into 60 slices according to the grid and processed for proteomics and complexome profiling analysis, related to **Fig 5f**. **f**, Summary of OxPhos complexes from complexome profiling of mitochondrial isolated from U2OS cells stably expressing *GFP* or *PI-PLC*^{IMS} in **Fig 5f**. Complex migration illustrated as a heatmap (top) or 2D complex profile (bottom). Abundance is normalized to the maximum across all gel slices and plotted versus slice number. For **a-b**, data are displayed as mean ± s.d., n=3 biologically independent samples, two-sided Student's *t*-test (for more than four comparisons *P<0.05, **P<0.01). Source data are provided as a Source Data file.

Supplementary References

1. Stefely, J. A. *et al.* Mitochondrial protein functions elucidated by multi-omic mass spectrometry profiling. *Nat Biotechnol* **34**, 1191–1197 (2016).

2. Rensvold, J. W. *et al.* Defining mitochondrial protein functions through deep multiomic profiling. *Nature* **606**, 382–388 (2022).
