# Supplementary material for: Fmp30p is a mitochondrial phosphatidylinositol hydrolase that modulates CoQ biosynthesis
Source: Nat Commun. 2026 May 30;17:7013. doi: 10.1038/s41467-026-73766-x (PMC13392021; doi:10.1038/s41467-026-73766-x)
Supplement: Supplementary file 2 — Reporting Summary [file 41467_2026_73766_MOESM2_ESM.pdf]

nature portfolio

Corresponding author(s): David Pagliarini

Last updated by author(s): Apr 29, 2026

Reporting Summary

Nature Portfolio wishes to improve the reproducibility of the work that we publish. This form provides structure for consistency and transparency in reporting. For further information on Nature Portfolio policies, see our Editorial Policies and the Editorial Policy Checklist.

Please do not complete any field with "not applicable" or n/a. Refer to the help text for what text to use if an item is not relevant to your study.
For final submission: please carefully check your responses for accuracy; you will not be able to make changes later.

Statistics

For all statistical analyses, confirm that the following items are present in the figure legend, table legend, main text, or Methods section.

n/a	Confirmed	
☐	☒	The exact sample size (n) for each experimental group/condition, given as a discrete number and unit of measurement
☐	☒	A statement on whether measurements were taken from distinct samples or whether the same sample was measured repeatedly
☐	☒	The statistical test(s) used AND whether they are one- or two-sided<br>*Only common tests should be described solely by name; describe more complex techniques in the Methods section.*
☒	☐	A description of all covariates tested
☒	☐	A description of any assumptions or corrections, such as tests of normality and adjustment for multiple comparisons
☐	☒	A full description of the statistical parameters including central tendency (e.g. means) or other basic estimates (e.g. regre ssion coefficient) AND variation (e.g. standard deviation) or associated estimates of uncertainty (e.g. confidence intervals)
☐	☒	For null hypothesis testing, the test statistic (e.g. F, t, r) with confidence intervals, effect sizes, degrees of freedom and P value noted<br>*Give P values as exact values whenever suitable.*
☒	☐	For Bayesian analysis, information on the choice of priors and Markov chain Monte Carlo settings
☒	☐	For hierarchical and complex designs, identification of the appropriate level for tests and full reporting of outcomes
☒	☐	Estimates of effect sizes (e.g. Cohen's d, Pearson's r), indicating how they were calculated

Our web collection on statistics for biologists contains articles on many of the points above.

Software and code

Policy information about availability of computer code

Data collection	LC-MS data was collected using Xcalibur (Thermo Scientific).<br>Growth assay data was collected using Gen5 v3.03.3 (BioTek).<br>Fluorescent Microscopy images were acquired with using Nikon-NIS Elements v5.21.00 (Nikon)
Data analysis	LC-MS targeted lipidomics was analyzed by Tracefinder 5.1 (Thermo)<br>LC-MS untargeted lipidomics was analyzed by Lipidex v2.0<br>LC_MS proteomic files were analyzed using Proteome Discoverer v2.5 and v3.2 (Thermo)<br>LC-MS lipidomics was analyzed using Compound Discoverer v3.3 (Thermo)<br>Fluorescent images were analyzed using ImageJ software v1.45v<br>Statistical analysis and all graphing was performed using python v3.10

For manuscripts utilizing custom algorithms or software that are central to the research but not yet described in published literature, software must be made available to editors and reviewers. We strongly encourage code deposition in a community repository (e.g. GitHub). See the Nature Portfolio guidelines for submitting code & software for further information.

Data

Policy information about availability of data

All manuscripts must include a data availability statement. This statement should provide the following information, where applicable:

- Accession codes, unique identifiers, or web links for publicly available datasets
- A description of any restrictions on data availability
- For clinical datasets or third party data, please ensure that the statement adheres to our policy

All mass spectrometry raw files have been deposited to MassIVE repository (accession number MSV000100896). All other relevant data are present in the Source Data files.

Research involving human participants, their data, or biological material

Policy information about studies with human participants or human data. See also policy information about sex, gender (identity/presentation), and sexual orientation and race, ethnicity and racism.

Reporting on sex and gender	n/a
Reporting on race, ethnicity, or other socially relevant groupings	n/a
Population characteristics	n/a
Recruitment	n/a
Ethics oversight	n/a

Note that full information on the approval of the study protocol must also be provided in the manuscript.

Field-specific reporting

Please select the one below that is the best fit for your research. If you are not sure, read the appropriate sections before making your selection.

☒ Life sciences ☐ Behavioural & social sciences ☐ Ecological, evolutionary & environmental sciences

For a reference copy of the document with all sections, see nature.com/documents/nr-reporting-summary-flat.pdf

Life sciences study design

All studies must disclose on these points even when the disclosure is negative.

Sample size	No statistical methods were used to predetermine sample size. All experiments were performed in at least biological triplicate, which is a generally accepted standard for the minimum number of replicates needed to obtain conclusive evidence for these types of experiments.
Data exclusions	No data were excluded from these analyses.
Replication	All attempts at experimental replication were successful. All experiments were performed in at least biological triplicate, as indicated in the figure legends.
Randomization	Randomization of experimental groups was not relevant. Quantitative measurements were measured by machine, mitigating investigator bias.
Blinding	Blinding of experimental groups was not relevant as experimental measurements were generated by automated measurements or computational analyses.

Reporting for specific materials, systems and methods

We require information from authors about some types of materials, experimental systems and methods used in many studies. Here, indicate whether each material, system or method listed is relevant to your study. If you are not sure if a list item applies to your research, read the appropriate section before selecting a response.

Materials & experimental systems

n/a	Involved in the study
☐	☒ Antibodies
☐	☒ Eukaryotic cell lines
☒	☐ Palaeontology and archaeology
☒	☐ Animals and other organisms
☒	☐ Clinical data
☒	☐ Dual use research of concern
☒	☐ Plants

Methods

n/a	Involved in the study
☒	☐ ChIP-seq
☒	☐ Flow cytometry
☒	☐ MRI-based neuroimaging

Antibodies

Antibodies used	Primary antibodies include anti-Kar2 (SCBT sc-33630), and anti-Cit1 (custom made at Biomatik), anti-Por1 (Abcam ab110326), anti-Pma1 (Abcam ab4645), and anti-NeonGreen (Proteintech 29523-1-AP). Secondary antibodies include anti-mouse immunoglobulin-G (IgG) horseradish peroxidase (HRP)-linked (Cell Signaling Technology #7076) and anti-rabbit IgG HRP-linked (Cell Signaling Technology #7074).
Validation	All antibodies were validated based on the manufacturer's website and literature support. For each immunoblot, we confirmed that the detected band migrated to the expected size of the protein products.

Eukaryotic cell lines

Policy information about cell lines and Sex and Gender in Research

Cell line source(s)	Saccharomyces cerevisiae haploid strain W303 (MATa leu2 trp1 can1 ura3 ade2 his3) (from Jared Rutter, University of Utah) and BY4742 (Mata leu2 his3 lys2 ura3) (from Betty Craig, University of Wisconsin-Madison). All single and double gene disruption strains were generates from this parent wild-type strain.<br>U2OS cells from ATCC (HTB-96)
Authentication	S. cerevisiae gene disruptions were confirmed by PCR assay. All strains carrying auxotrophic markers were cultured under selective conditions. U2OS cell line was authenticated by ATCC.
Mycoplasma contamination	All cell lines were negative for mycoplasma contamination as tested using a commercial test kit.
Commonly misidentified lines (See ICLAC register)	No commonly misidentified lines were used.

Plants

Seed stocks	n/a
Novel plant genotypes	n/a
Authentication	n/a

This checklist template is licensed under a Creative Commons Attribution 4.0 International License, which permits use, sharing, adaptation, distribution and reproduction in any medium or format, as long as you give appropriate credit to the original author(s) and the source, provide a link to the Creative Commons license, and indicate if changes were made. The images or other third party material in this article are included in the article's Creative Commons license, unless indicated otherwise in a credit line to the material. If material is not included in the article's Creative Commons license and your intended use is not permitted by statutory regulation or exceeds the permitted use, you will need to obtain permission directly from the copyright holder. To view a copy of this license, visit http://creativecommons.org/licenses/by/4.0/
