# Supplementary material for: Fmp30p is a mitochondrial phosphatidylinositol hydrolase that modulates CoQ biosynthesis
Source: Nat Commun. 2026 May 30;17:7013. doi: 10.1038/s41467-026-73766-x (PMC13392021; doi:10.1038/s41467-026-73766-x)

Unprocessed immunoblots

Related to: Supplementary Fig. 3a

Published figure panel

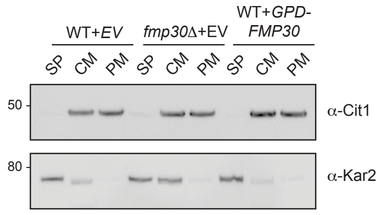

Raw images

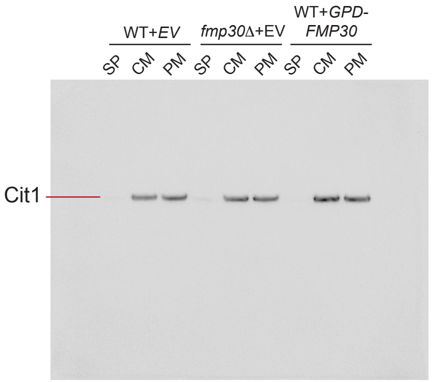

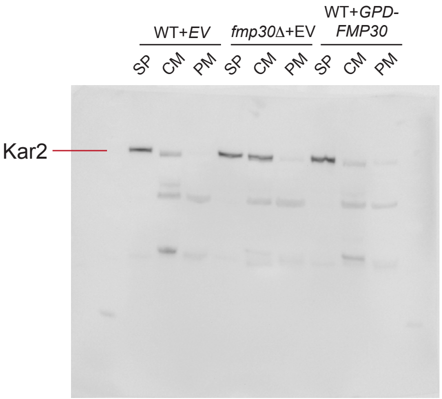

Unprocessed immunoblots

Related to: Supplementary Fig. 4g

Published figure panel

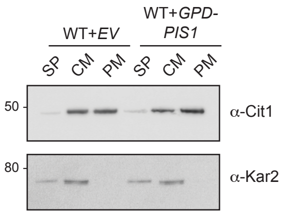

Raw images

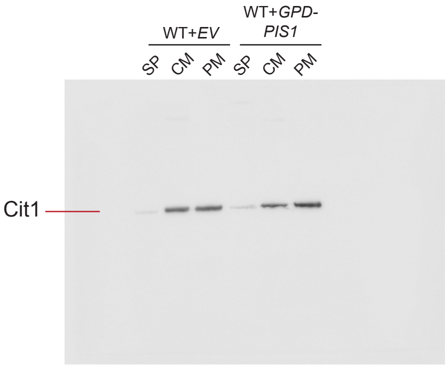

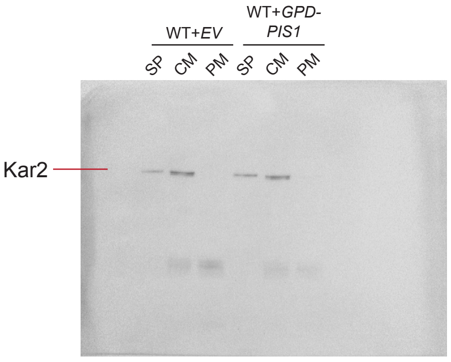

Unprocessed immunoblots

Related to: Supplementary Fig. 4z

Published figure panel

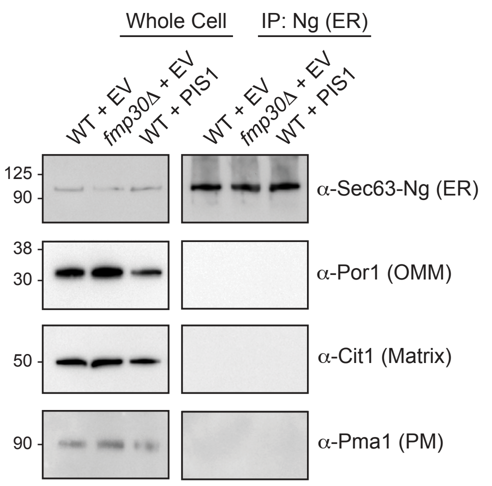

Raw images

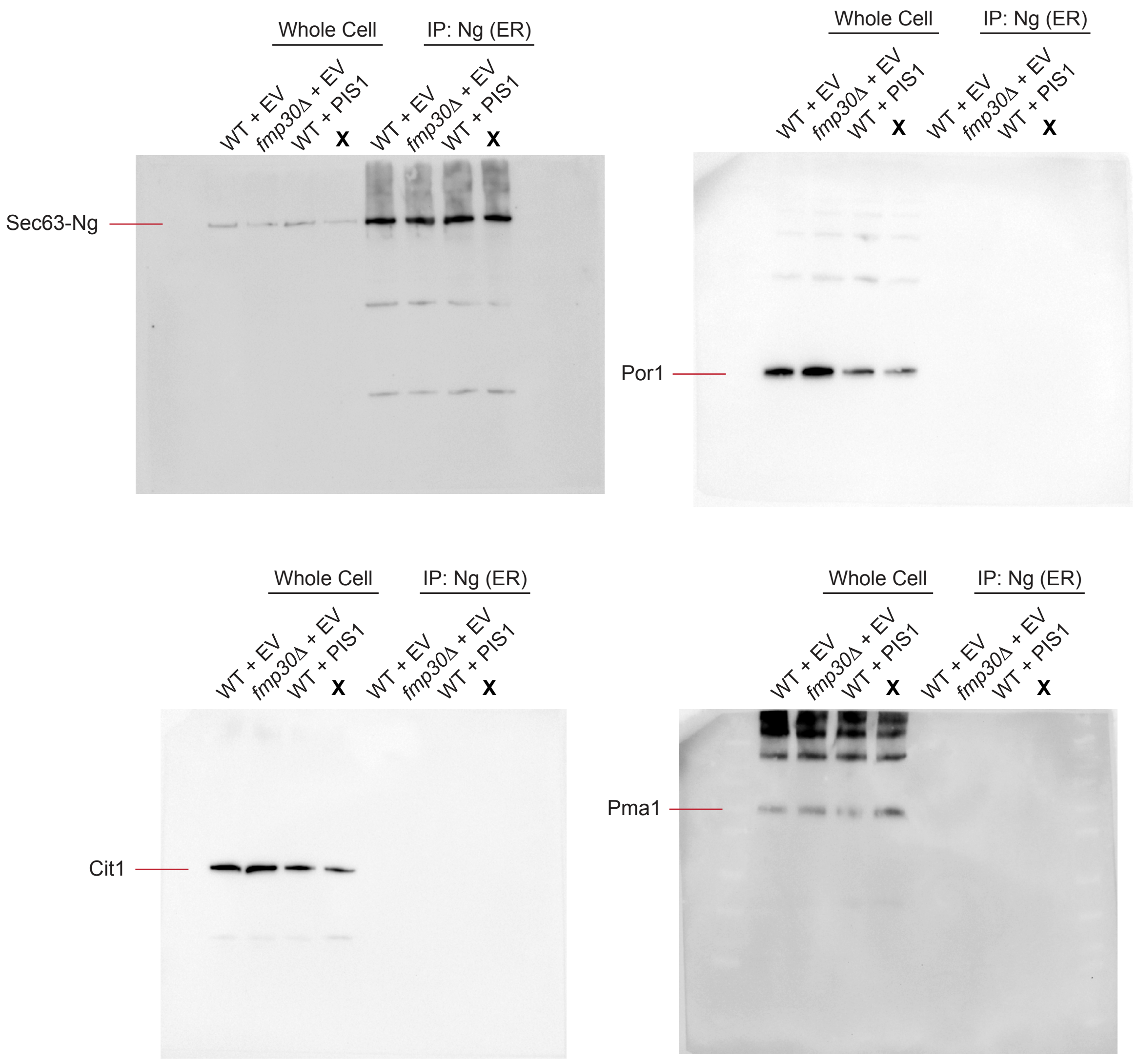

Unprocessed immunoblots

Related to: Supplementary Fig. 3g

Published figure panel

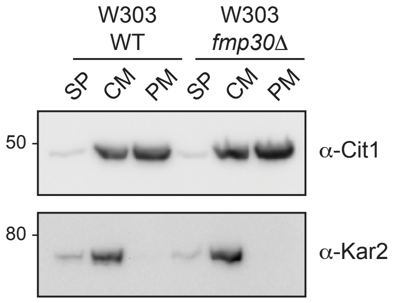

Raw images

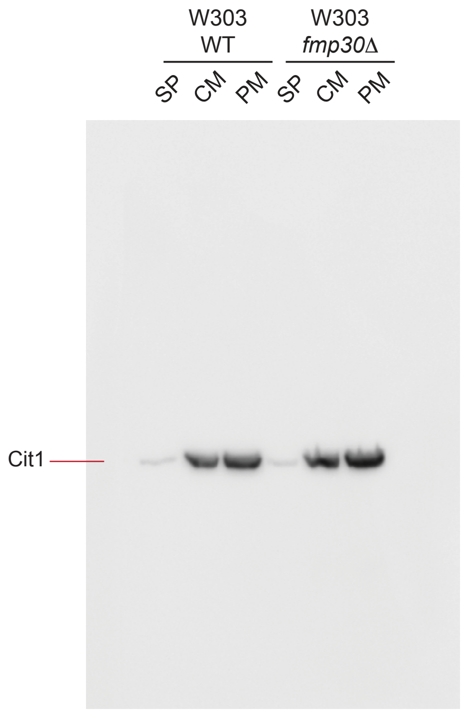

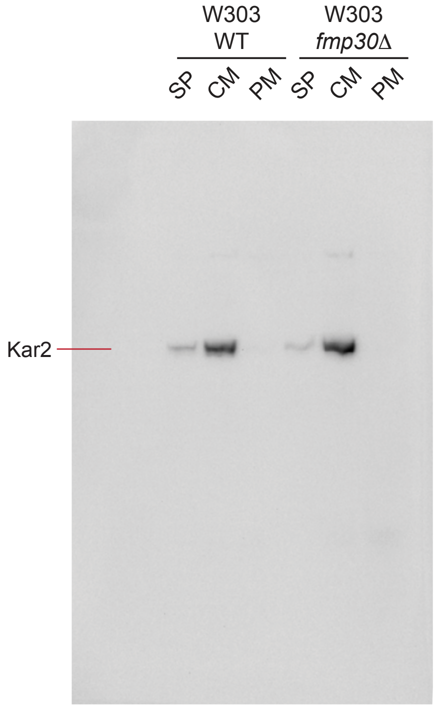

Unprocessed immunoblots

Related to: Supplementary Fig. 2b-d

Published figure panel a

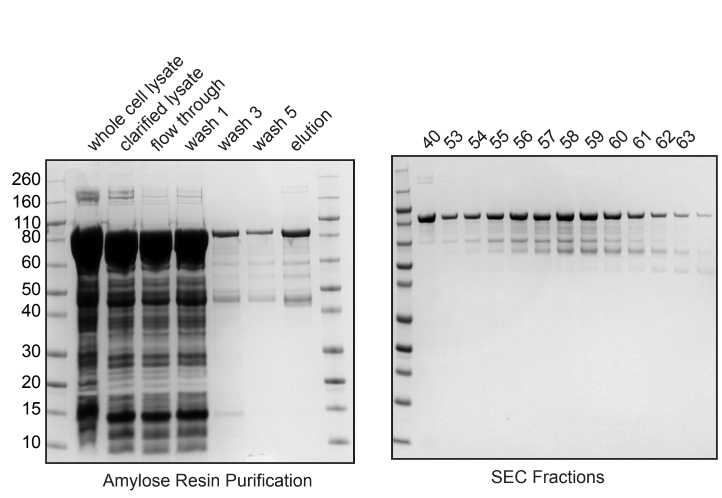

Raw images

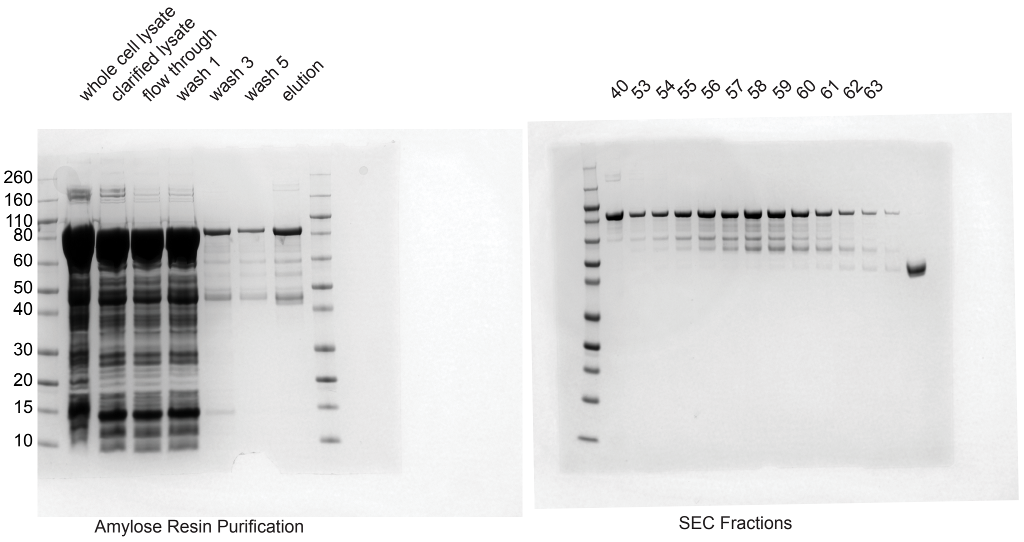

Published figure panel b

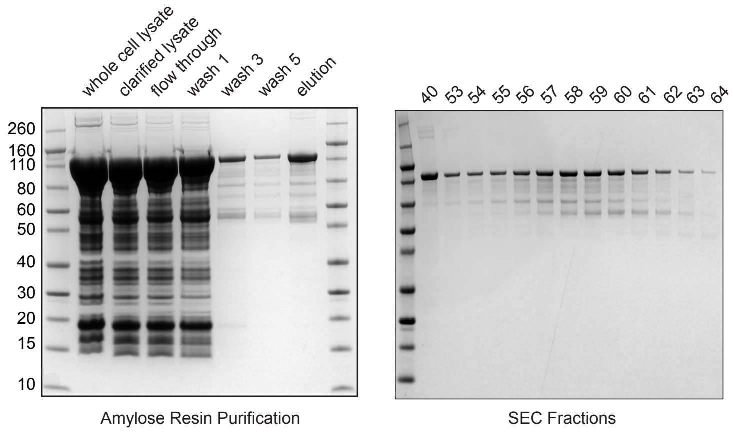

Raw images

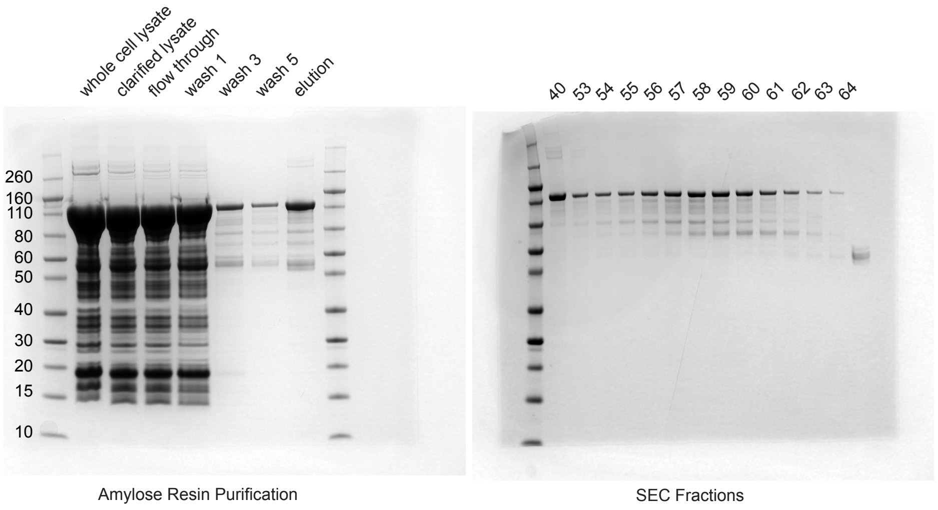

Published figure panel c

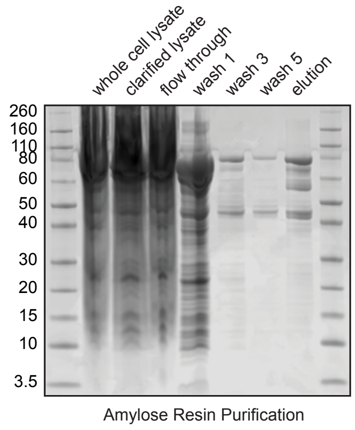

Raw images

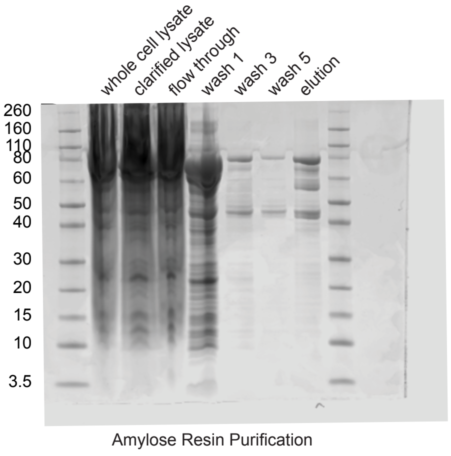

Supplement: Supplementary file 4 — Source Data [file 41467_2026_73766_MOESM4_ESM.zip › 2026_Baker_Guerra_Nat_Comm_Unprocessed_Immunoblots.pdf]
